# The Systematic Investigation of the Quorum Sensing System of the Biocontrol Strain *Pseudomonas chlororaphis* subsp. *aurantiaca* PB-St2 Unveils *aurI* to Be a Biosynthetic Origin for 3-Oxo-Homoserine Lactones

**DOI:** 10.1371/journal.pone.0167002

**Published:** 2016-11-18

**Authors:** Judith S. Bauer, Nils Hauck, Lisa Christof, Samina Mehnaz, Bertolt Gust, Harald Gross

**Affiliations:** 1 Department of Pharmaceutical Biology, Pharmaceutical Institute, University of Tuebingen, Tuebingen, Germany; 2 German Centre for Infection Research (DZIF), Partner site Tuebingen, Tuebingen, Germany; 3 Department of Biological Sciences, Forman Christian College (A Chartered University), Lahore, Pakistan; East Carolina University Brody School of Medicine, UNITED STATES

## Abstract

The shoot endophytic biocontrol strain *Pseudomonas chlororaphis* subsp. *aurantiaca* PB-St2 produces a wide range of exoproducts, including enzymes and antibiotics. The production of exoproducts is commonly tightly regulated. In order to get a deeper insight into the regulatory network of PB-St2, the strain was systematically investigated regarding its quorum sensing systems, both on the genetic and metabolic level. The genome analysis of PB-St2 revealed the presence of four putative acyl homoserine lactone (AHL) biosynthesis genes: *phzI*, *csaI*, *aurI*, and *hdtS*. LC-MS/MS analyses of the crude supernatant extracts demonstrated that PB-St2 produces eight AHLs. In addition, the concentration of all AHL derivatives was quantified time-resolved in parallel over a period of 42 h during the growth of *P*. *aurantiaca* PB-St2, resulting in production curves, which showed differences regarding the maximum levels of the AHLs (14.6 nM– 1.75 μM) and the production period. Cloning and heterologous overexpression of all identified AHL synthase genes in *Escherichia coli* proved the functionality of the resulting synthases PhzI, CsaI, and AurI. A clear AHL production pattern was assigned to each of these three AHL synthases, while the HdtS synthase did not lead to any AHL production. Furthermore, the heterologous expression study demonstrated unequivocally and for the first time that AurI directs the synthesis of two 3-oxo-AHLs.

## Introduction

Due to its use as raw material for food industry and biofuel production, sugarcane (*Saccharum* sp. hybrids) represents an economically important crop in South Asia, Melanesia, and Central- and South America. Numerous pathogens generate significant yield losses [1] and a major contributor thereof represents the fungus *Colletotrichum falcatum* that causes the red rot disease [2]. This disease is difficult to eradicate as disease outbreaks can continue to occur across several seasons, often originating from dormant spores. Due to the devastating and lasting impact of *C*. *falcatum*, the preventive application of agrochemicals is a common practice. However, since the regular use of fungicides worsens the resistance situation, poses a risk to the environment and the long-term fertility of the soil [3–7], biological control agents such as plant growth promoting rhizobacteria are gaining a considerable interest as an inroad to overcome these problems. In order to find a *Colletotrichum*-active biocontrol strain, a ´suppressive soil´-approach [8, 9] was employed. Thirty-two bacterial strains were isolated from the root, shoot, and rhizosphere of disease-tolerant sugarcane plants and screened for their antagonistic activity towards *C*. *falcatum*. From this screening, *Pseudomonas chlororaphis* subsp. *aurantiaca* PB-St2 had been selected as a promising biocontrol agent [10]. Its antifungal activity was so far attributed to the production of phenazine-1-carboxylic acid and 2-hydroxyphenazine [11]. Furthermore, strain PB-St2 was shown to produce three aromatic acids called lahorenoic acids A-C, 2-hydroxyphenazine-1-carboxylic acid, 2,8-dihydroxyphenazine, the lipopeptide WLIP, hydrogen cyanide, and C6-homoserine lactone (HSL) [11, 12]. Genome sequencing of PB-St2 revealed that the strain possesses in addition the biosynthetic capacity to produce the antifungal metabolite pyrrolnitrin, and siderophores of the achromobactin and pyoverdin-type [13].

The compound class of acyl homoserine lactones (AHLs), are canonical quorum sensing (QS) signal molecules which are typically produced by many proteobacteria [14]. AHLs regulate in a cell-density-dependent manner a variety of physiological processes, such as bioluminescence [15], swarming and swimming motility [16, 17], biofilm differentiation and development [18–22], secondary metabolite production [23–26], conjugation and virulence gene expression (for reviews see Ref. [27–31]). It has been found that proteins belonging to two families are essential: LuxI (AHL synthase) and LuxR (AHL receptor). LuxI synthesizes AHLs that bind after reaching a certain threshold to the cytoplasmic receptor protein LuxR and thus prevent it from degradation. Subsequently, this receptor regulates the transcription of target genes [32–35].

**Fig 1:**
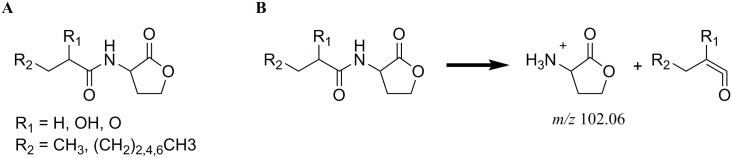
Structural formulas and MS/MS fragmentation of AHLs. (A) Structural formulas of AHLs produced by *P*. *aurantiaca* PB-St2. (B) Fragmentation of AHLs into the [M+H]^+^ ion of the lactone moiety in the collision cell.

AHLs consist of a five-membered homoserine lactone ring with an amide-linked side-chain. The side chain is varying in length (C4-C18), in the nature of the substitution at the carbon atom at position-3 (no substitution, keto-, or hydroxyl-group), and in its degree of saturation [27] (Fig 1A). Empirically, if proteobacteria employ an AHL cell to cell communication system, they produce a suite of AHLs, but with one principle component [36]. However, for PB-St2, evidence was so far supplied only for the production of C6-HSL using a TLC overlay experiment with *Chromobacterium violaceum* CV026 [12]. Furthermore, AHL-based QS systems are known to be of great importance for many plant-associated biocontrol strains, because QS systems control the production of antibiotics [37–40]. Since the genome sequencing analysis revealed that strain PB-St2 possesses at least three QS systems [13], we began to explore the possibility that PB-St2 produces a variety of AHLs and that AHLs may be involved in regulating the biocontrol properties of PB-St2. Therefore, we sought to (i) identify and chemically characterize all AHLs produced by PB-St2, and (ii) clone and heterologously overexpress genes involved in AHL biosynthesis to prove their function. In this study, we (a) showed that PB-St2 possesses indeed four putative AHL synthases, (b) demonstrated that three thereof produced eight different AHLs in different concentrations in a time-dependent manner, (c) clarified the biosynthetic origin of each AHL compound, and (d) thereby discovered the biosynthetic origin for 3-oxo-AHLs.

## Materials and Methods

### Bacterial strains and growth conditions

Bacterial strains and plasmids used in this study are described in Table 1. All strains of *Escherichia coli* were grown in lysogeny broth (LB) at 37°C. Antibiotics were added to final concentrations of 50 μg/mL carbenicillin (carb) and 25 μg/mL tetracycline (Tc). For blue/white screening, IPTG (isopropyl-β-D-thiogalactopyranoside) and X-Gal (5-bromo-4-chloro-3-indolyl-β-D-galactopyranoside) were added to a final concentration of 0.1 mM and 120 μg/mL, respectively.

**Table 1:** Bacterial strains, plasmids, and primers used in this study.

Strain or plasmid	Relevant characteristics	Reference or source
**Strains**		
*P*. *chlororaphis* subsp. *aurantiaca* PB-St2	wild type strain	[13]
*E*. *coli* XL1-Blue	*rec*A1 *end*A1 *gyr*A96 *thi*-1 *hsd*R17 *sup*E44 *rel*A1 *lac* [F´ *pro*AB *lac*I^q^ ZΔM15 Tn10 (Tet^r^)]	Stratagene
**Plasmids**		
pBluescript SK(-)	Cloning vector (phagemid excised from lambda ZAP). The f1 (–) orientation allows rescue of antisense strand ssDNA	Stratagene
pNH07	pBluescript SK(-) containing the inducible *lac*-promotor and *aurI* from *P*. *aurantiaca* PB-St2 at PsiI-PciI sites	This study
pNH08	pBluescript SK(-) containing the inducible *lac*-promotor and *csaI* from *P*. *aurantiaca* PB-St2 at PsiI-PciI sites	This study
pNH09	pBluescript SK(-) containing the inducible *lac*-promotor and *phzI* from *P*. *aurantiaca* PB-St2 at PsiI-PciI sites	This study
pNH10	pBluescript SK(-) containing the inducible *lac*-promotor and *hdtS* from *P*. *aurantiaca* PB-St2 at PsiI-PciI sites	This study
pNH11	pBluescript SK(-)Δ*lacZ* cut at PsiI-EcoRV sites	This study
pNH12	pBluescript SK(-) containing the inducible *lac*-promotor and *aurI* from *P*. *chlororaphis* subsp. *aurantiaca* StFRB508 at PsiI-PciI sites	This study
pNH13	pBluescript SK(-) containing the inducible *lac*-promotor and *hdtS* from *P*. *fluorescens* F113 at PsiI-PciI sites	This study

### Bioinformatic analysis

The genome sequence was analyzed using antiSMASH 3.0 software to identify putative QS systems in *P*. *aurantiaca* PB-St2 [41]. In addition, for a detailed analysis of AHL synthases, AHL acylases, and other QS related genes manual BLAST searches were performed using Bio-Edit [42]. Protein sequence alignments were performed using EMBOSS Needle [43].

### Gene design

The coding sequences of aurI, csaI, phzI, and hdtS from P. aurantiaca PB-St2, the coding sequence of aurI_StFRB508 from P. chlororaphis subsp. aurantiaca StFRB508 and the coding sequence of hdtS_F113 from P. fluorescens F113, all with a PciI site and under control of the IPTG-inducible lac-promoter were ordered as gBlocks^®^ (IDT, sequence see S1–S3 Tables). The genes aurI and aurI_StFRB508 were codon optimized in two positions at the end due to a repetitive sequence (ATC AGC GCC TGA →ATT AGC GCA TGA).

### Plasmid constructions

Standard methods were used for plasmid DNA isolation, restriction enzyme digestion, agarose gel electrophoresis, ligation, and transformation [44]. The ordered gBlocks^®^ and pBluescript SK (-) (Stratagene, La Jolla, CA) were digested with PciI and PsiI. Fragment sizes were confirmed by gel electrophoresis and the product sizes of 1,022 bp (*aurI*), 1,022 bp (*aurI*_StFRB508), 1,001 bp (*csaI*), 1,112 bp (*hdtS*), 1,121 bp (*hdtS*_F113), 932 bp (*phzI*), and 2,170 bp (pBluescript SK (-)) were purified using preparative gel electrophoresis and the peqGOLD Gel Extraction Kit (PeqLab). The fragments were cloned into pBluescript SK (-) to give plasmids pNH07—pNH10 and pNH12-pNH13, respectively.

As a negative control pBluescript SK (-) was digested with EcoRV and PsiI. Fragments were confirmed by gel electrophoresis and the product size of 2,626 bp was purified using preparative gel electrophoresis and the peqGOLD Gel Extraction Kit (PeqLab). Religation of the vector resulted in pNH11, containing the *lac*-promotor, but missing *lacZ*.

All constructs were transformed into *E*. *coli* XL1-Blue (Stratagene) using electroporation, and selected with carb, Tc, and blue/white selection.

### Plasmid confirmation

White clones were screened by colony-PCR with primers (5´→ 3´) pBluescript_f (AGTGCTTTACGGCACCTCGAC) and pBluescript_r (GCCACCTCTGACTTGAGCGTC). The PCR temperature profile was as follows: 5 min at 94°C, followed by 30 cycles of 45 s at 94°C, 45 s at 61°C, and 60 s at 72°C, and a final extension step of 5 min at 72°C. PCR-products were confirmed by gel electrophoresis with a predicted size of 1,319 bp (*aurI*), 1,319 bp (*aurI*_StFRB508), 1,298 bp (*csaI*), 1,409 bp (*hdtS*), 1,418 bp (*hdtS*_F113), and 1,229 bp (*phzI*). In addition, positive clones were verified by DNA sequence analysis.

### Identification of AHLs produced by *P*. *aurantiaca* PB-St2

For initial inoculums *P*. *aurantiaca* PB-St2 was grown for two days in 8 mL LB at 25°C and 130 rpm. For main cultures, LB was supplemented with 100 mM phosphate buffer (pH 6.5) to prevent degradation of the AHLs due to alkaline pH. Subsequently, 50 mL medium in 300 mL Erlenmeyer flasks was inoculated with 100 μL initial inoculum and cultivated at 25°C and 130 rpm. After one day of growth, 40 mL of the culture was extracted, as described previously [38]. Briefly, culture supernatants were extracted (1:1) with ethyl acetate, acidified with 0.1% acetic acid. The solvent phase was evaporated under a stream of nitrogen and the extract resolved in 300 μL acetonitrile (LC-MS grade, Sigma Aldrich) for LC-MS analysis. The AHLs were identified via comparison of the retention time and the fragmentation pattern with commercially available reference standards (University of Nottingham, Sigma Aldrich, Santa Cruz Biotechnology) and coinjection (1:1) of the standards and the crude extract.

### Identification of AHLs produced by the heterologous hosts

LB (50 mL) containing 100 mM phosphate buffer (pH 6.5) and a final concentration of 50 μg/mL carb in 300 mL Erlenmeyer flasks was inoculated to an optical density at 600 nm (OD_600_) of 0.05 with overnight cultures of *E*. *coli* XL1-Blue/pNH07-13. Cultures were grown at 37°C with 200 rpm shaking until an OD_600_ of 0.5 was reached. Expression of AHL synthases was induced by adding IPTG to a final concentration of 1 mM. Subsequently, the temperature was lowered to 25°C. After one day, 40 mL of the cultures were extracted, as described above.

### LC-MS/MS analyses

For LC-MS/MS analyses an 1100 Series HPLC system (Agilent Technologies, Waldbronn, Germany) was used. The Agilent HPLC components (G1322A degasser, G1312A binary pump, G1329A autosampler, G1315A diode array detector) were connected with an ABSCIEX 3200 Q TRAP LC/MS/MS mass spectrometer (AB Sciex, Germany GmbH, Darmstadt, Germany). For measurements, the following LC gradient was used: Starting with two minutes H_2_O acidified with 0.025% formic acid (solvent A):acetonitrile (solvent B, LC-MS grade, Sigma Aldrich) (95:5) followed by a gradient to A:B (5:95) in 30 min, flow rate 0.6 mL/min; injection volume 20 μL; column: Waters Symmetry Shield RP18 (5 μm, 250×4.6 mm, Waters GmbH, Eschborn, Germany). MS measurements were performed in positive ionization mode. For precursor ion scan measurements, parameters were optimized for C6-HSL using the “automatic compound optimization” option of the Analyst LC/MS software (AB Sciex, Germany GmbH). As precursor *m/z* 102.1 was used. The following parameters were optimized: curtain gas 10 psi, temperature 450°C, gas 1 and 2 20 psi, ion spray voltage 5,500 V, declustering potential 46 V, collision energy 13 V, entrance potential 12 V, scan area 50–400 Da. To obtain MS/MS product ion spectra of the AHLs, MS parameters were optimized separately, for each standard.

### Quantification of the AHLs

For quantification of the AHLs, *P*. *aurantiaca* PB-St2 cultures were grown and extracted, as described above. To obtain production curves, cultures were extracted at a two hour interval between 5 h to 27 h, and after 42 h. At each time point, pH and the OD_600_ value of the culture were measured. A C9-HSL solution with a concentration of 0.5 μg/mL was added 1:1 to every sample as an internal standard. To screen for linearity solutions of C4-HSL, C6-HSL, C8-HSL, 3-OH-C6-HSL, 3-OH-C8-HSL, 3-OH-C10-HSL, 3-oxo-C6-HSL, and 3-oxo-C8-HSL with concentration between 10 ng/mL and 1 mg/mL were measured, employing the LC-MS/MS precursor ion scan mode. For each AHL, a five-point calibration curve with equidistant concentrations was constructed, beginning at the lowest possible detection point (S8 Fig). Limits of quantification are listed in S2 Table. Samples were diluted to reach a concentration of AHL that was localized approximately in the middle of the corresponding calibration curve and the amount of the AHL was calculated using the linear calibration equation (S4 Table).

### Monitoring for phenazine production

Phenazine production of *P*. *aurantiaca* PB-St2 was monitored at a two hour interval between 5 h and 27 h, and after 42 h. Therefore, 1 mL culture was centrifuged; the supernatant was acidified with 100 μL 1 N HCl and extracted with 1 mL chloroform. To estimate the amount of phenazines, the absorption at 365 nm was measured with a Lambda 25 UV/VIS spectrometer (Perkin Elmer). A 2 mm glass cuvette was used and solutions were diluted when necessary. The A_365_ value represents the amount of phenazines in 1 mL culture.

## Results

### Identification of the QS systems in *P*. *aurantiaca* PB-St2

In order to identify all putative QS systems in *P*. *aurantiaca* PB-St2, the whole genome sequence [13] was analyzed using antiSMASH 3.0 software, which readily identified three putative QS systems. To obtain more information about these systems, a BLAST search for already known and characterized systems was performed. Two of the identified QS systems showed 93%/93% and 95%/97% nucleotide sequence identity to *P*. *chlororaphis* subsp. *aureofaciens* 30–84 genes *phzI/R* and *csaI/R*, respectively [36]. The third QS system revealed 96% and 97% sequence identity to the QS genes *aurI* and *aurR* from *P*. *chlororaphis* subsp. *aurantiaca* StFRB508 [45]. Manual BLAST searches led to the detection of a fourth AHL synthase with 82% sequence identity to *hdtS* from *P*. *fluorescens* F113 [37]. Thus, in total four different putative AHL synthases were detected in the genome of *P*. *aurantiaca* PB-St2 (S1 Fig).

A manual BLAST search for the quinolone biosynthetic gene cluster *pqsA-E* and for 2-(2-hydroxyphenyl)-thiazole-4-carbaldehyde (IQS) biosynthetic gene cluster *ambBCDE* from *Pseudomonas aeruginosa* PAO1 [46–48] revealed no homologous genes. This indicated that *P*. *aurantiaca* PB-St2 is only using AHL-based QS and no quinolone-based or IQS-based cell to cell communication systems [49].

### Chemical characterization of AHLs produced by *P*. *aurantiaca* PB-St2

In order to get insight into the complete spectrum of AHL-based compounds, produced by PB-St2, corresponding extracts were analyzed using LC-MS/MS in the precursor ion scan mode. In this mode the *m/z* of all ions that lead to the same fragment in the collision cell were recorded. The [M+H]^+^ ion of the AHLs decomposed to the [M+H]^+^ ion of the lactone moiety at *m/z* 102 (Fig 1B) [50]. Since this fragment is independent of the length and the substituent at position-3 of the acyl moiety, it was used as the precursor ion to identify the mass of bacterial AHLs.

**Fig 2:**
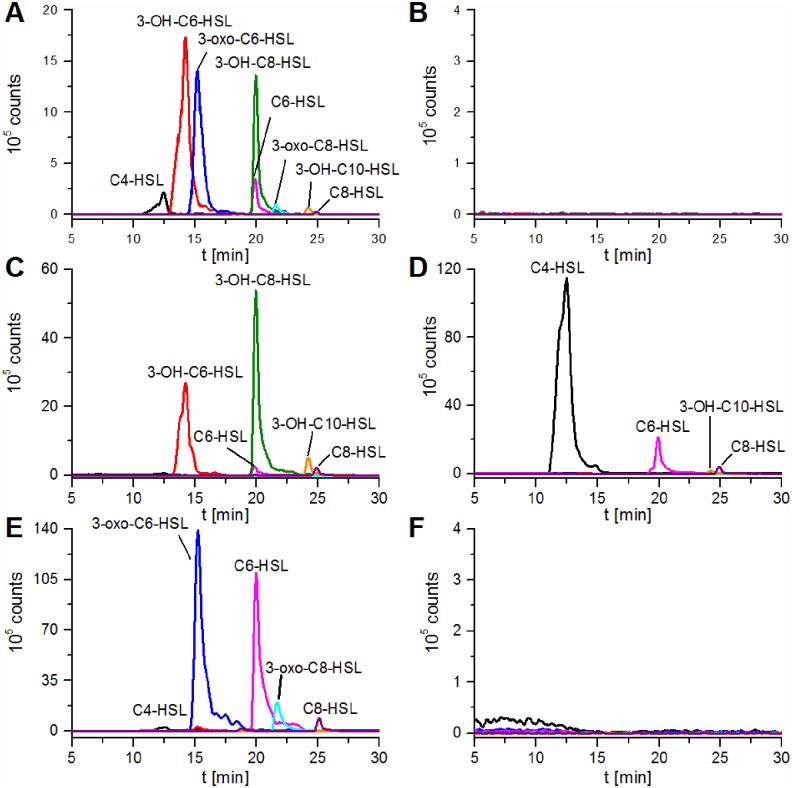
Identification of the produced AHLs using LC-MS/MS. Extracted ion chromatograms (LC-MS/MS, precursor ion scan, positive ionization mode) of [M+H]^+^ ions of AHLs present in (A) *P*. *aurantiaca* PB-St2 crude extract after 22 h cultivation, (B) *E*. *coli* XL1-Blue/pBluescript SK(-)Δ*lacZ* crude extract, and extracts of *E*. *coli* XL1-Blue expressing (C) *phzI*, (D) *csaI*, (E) *aurI*, and (F) *hdtS*. C4-HSL (black, *m/z* 172–173), 3-OH-C6-HSL (red, *m/z* 216–217), 3-oxo-C6-HSL (blue, *m/z* 214–215), C6-HSL (pink, *m/z* 200–201), 3-OH-C8-HSL (green, *m/z* 244–245), 3-oxo-C8-HSL (cyan, *m/z* 242–243), 3-OH-C10-HSL (orange, *m/z* 272–273), C8-HSL (purple, *m/z* 228–229).

Using this approach, eight peaks were identified that were absent in non-inoculated LB extracts (used as negative control) and occurred from [M+H]^+^ ions, corresponding to common AHLs (Fig 2A). To confirm the identity of the AHLs, the *P*. *aurantiaca* PB-St2 extract was compared with commercially available AHL standards. The peaks were identified as C4-HSL, 3-OH-C6-HSL, 3-oxo-C6-HSL, C6-HSL, 3-OH-C8-HSL, 3-oxo-C8-HSL, 3-OH-C10-HSL, and C8-HSL by comparison of retention times and coinjection in the precursor ion mode (S2 Fig) and corroborated by comparison of retention times and fragmentation patterns using an LC-MS/MS product ion method (S3 and S4 Figs).

Thus, in total eight AHLs were detected in the crude extract of PB-St2, but homologues of the AHL synthases of other pseudomonads lead in total to the production of ten different AHLs (Table 2) [36, 37, 45]. Due to the high sequence identity of the AHL synthases it was expected that *P*. *aurantiaca* PB-St2 is producing almost the same AHLs as listed in Table 2. However, iin comparison with the AHLs originating from AHL synthases with established functions, C5-HSL, 3-OH-C7-HSL, C10-HSL, and 3-OH-C14:1-HSL were not detected in *P*. *aurantiaca* PB-St2. Instead of these four AHLs, *P*. *aurantiaca* PB-St2 produced 3-oxo-AHLs, i.e., 3-oxo-C6- and 3-oxo-C8-HSL which were, so far, not known to be produced by any of the four AHL synthases. This raised two questions: First, since none of the *in silico* detected gene clusters were so far described to produce 3-oxo-AHLs, which AHL synthase system in PB-St2 is responsible for the production of the 3-oxo-AHLs. Second, considering that two typical AHL compounds that are commonly produced by HdtS were not detected in *P*. *aurantiaca* PB-St2, we questioned whether all four AHL synthases were actually functional. These questions were subsequently addressed with the heterologous overexpression of each AHL synthase gene, employing synthetic biology.

**Table 2:** Comparison of AHLs produced in different studies by different AHL synthases.

AHL synthase	produced AHLs in other studies [references]	produced AHLs in this study
PhzI	C5-HSL, C6-HSL, C8-HSL, 3-OH-C6-HSL, 3-OH-C7-HSL, 3-OH-C8-HSL, 3-OH-C10-HSL [36]	C6-HSL, C8-HSL, 3-OH-C6-HSL, 3-OH-C8-HSL, 3-OH-C10-HSL
CsaI	C4-HSL, C5-HSL, C6-HSL [36]	C4-HSL, C6-HSL, C8-HSL, 3-OH-C10-HSL
AurI	C4-HSL, C6-HSL [45]	C4-HSL, C6-HSL, C8-HSL, 3-oxo-C6-HSL, 3-oxo-C8-HSL
HdtS	C6-HSL, C10-HSL, 3-OH-C14:1-HSL [37]	-

### Correlation of the detected AHL compounds with the corresponding candidate QS gene cluster

To identify the signals produced by each candidate gene, we cloned the synthetically generated AHL synthase genes separately into *E*. *coli* under control of a *lac*-promotor. The heterologous producers and the negative control were cultivated, the supernatants extracted, and the AHL profile of the crude extracts compared using LC-MS/MS in the precursor ion mode (Fig 2B–2F). As expected the negative control *E*. *coli* XL1-Blue/pBluescript SK(-)Δ*lacZ* is not producing any AHLs (Fig 2B). Thus, *E*. *coli* XL1-Blue is a feasible host for expressing the AHL synthases. The recombinant PhzI from PB-St2 catalyzed the production of five AHLs. Analysis by LC-MS/MS confirmed the production of C6-HSL, C8-HSL, 3-OH-C6-HSL, 3-OH-C8-HSL, and 3-OH-C10-HSL (Fig 2C). In comparison with the synthase PhzI from *P*. *chlororaphis* subsp. *aureofaciens* 30–84 [36], the synthase PhzI of PB-St2 was shown to produce only a subset of AHLs because it was not producing C5-HSL and 3-OH-C7-HSL (Table 2). It is noteworthy to mention that PB-St2 did not produce any AHL in detectable amounts containing a fatty acid side chain with an odd number of carbons. The fatty acid side chains used in AHL biosynthesis derive from fatty acid biosynthesis. Fatty acids with an odd number of carbons arise from the use of propionyl-CoA instead of acetyl-CoA as starter unit. In comparison to acetyl-CoA propionyl-CoA is used very rarely in fatty acid biosynthesis, consequently odd numbered AHL are uncommon [28, 51]. Thus, we assume that the AHL synthases of *P*. *aurantiaca* PB-St2 are not able to produce AHLs with an odd numbered fatty acid side chain as the precursor is not provided by the fatty acid biosynthesis, at least under the applied conditions. This is in accordance with the fact that CsaI from PB-St2 was not producing C5-HSL, as well. However, CsaI was producing C8-HSL and 3-OH-C10-HSL (Fig 2D) which were not detected for CsaI from *P*. *chlororaphis* subsp. *aureofaciens* 30–84 [36].

AurI produced the same AHLs as previously reported for AurI from *P*. *chlororaphis* subsp. *aurantiaca* StFRB508 and in addition, two further AHLs: 3-oxo-C6-HSL and 3-oxo-C8-HSL (Fig 2E). In comparison with AurI of StFRB508, AurI of PB-St2 additionally appears to be capable of producing oxo-AHLs at first sight. A sequence alignment of the AurI AHL synthases from *P*. *aurantiaca* PB-St2 and *P*. *chlororaphis* subsp. *aurantiaca* StFRB508 demonstrated that the two synthases differ only in eight amino acids, i.e. AAs at position 3, 8, 23, 91, 147, 165, 206, and 219 (see S6 Table). This raised the question if AurI from StFRB508 is possibly also able to produce 3-oxo-AHLs or if some of the eight amino acids are indeed decisive for the substrate specificity. A direct analysis of the produced AHL spectrum of *P*. *chlororaphis* subsp. *aurantiaca* StFRB508 was not possible, since the strain is not available anymore in the scientific community. Therefore, to address this question, the synthetically generated *aurI* gene from *P*. *chlororaphis* subsp. *aurantiaca* StFRB508 was cloned into *E*. *coli* under control of a *lac*-promotor. The analysis of the AHLs produced by AurI from StFRB508 revealed that it is producing exactly the same AHLs as AurI from PB-St2 (S6 Fig).

For the fourth predicted AHL synthase HdtS no AHLs were detected in the heterologous host (Fig 2F). This finding is in accordance with the fact that no AHLs which are typically produced by HdtS [37] were detected in *P*. *aurantiaca* PB-St2. To exclude possible failures in our *E*. *coli*-based expression system we heterologously expressed the synthetically generated *hdtS* gene from *P*. *fluorescens* F113, whose function as AHL synthase was validly established. As expected HdtS from F113 was producing C6-HSL, while for HdtS from PB-St2 no production was detected (S5 Fig). This supported the assumption that HdtS from *P*. *aurantiaca* PB-St2 is inactive.

### Time-dependent quantification of AHL production by LC-MS/MS

**Fig 3:**
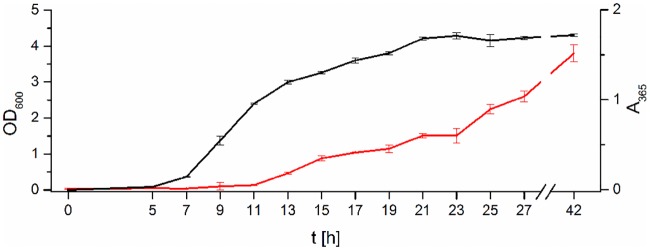
Growth curve of *P*. *aurantiaca* PB-St2 and production curve of phenazines. The growth curve of *P*. *aurantiaca* PB-St2 was measured as OD_600_ (black) and the production curve of phenazines as A_365_ (red). Data represent means with corresponding standard deviation of three independent replicates.

We next determined the concentration of each AHL produced by *P*. *aurantiaca* PB-St2 within a time range of 42 h to investigate at which point in time and at which amount the AHL production occurs. To correct changes in LC-MS instrument sensitivity, C9-HSL was used as an internal standard, because it was not produced by *P*. *aurantiaca* PB-St2 (S7 Fig). For quantification, equidistant five-point calibration curves for each AHL were constructed. The amount of AHLs produced between 5–27 hours and after 42 hours was determined by extracting the culture every two hours and measuring the crude extract using LC-MS/MS. Furthermore, a growth curve was determined (Fig 3). Since the detected *phzIR* genes in strain PB-St2 were located directly upstream of the *phzABCDEFGO* gene cluster (S1 Fig), they were anticipated to regulate phenazine production [36, 52]. Therefore, the production of phenazines was monitored in parallel by measuring A_365_ (Fig 3). Phenazine production started after 13 hours of growth and increased continuously until 42 hours.

**Fig 4:**
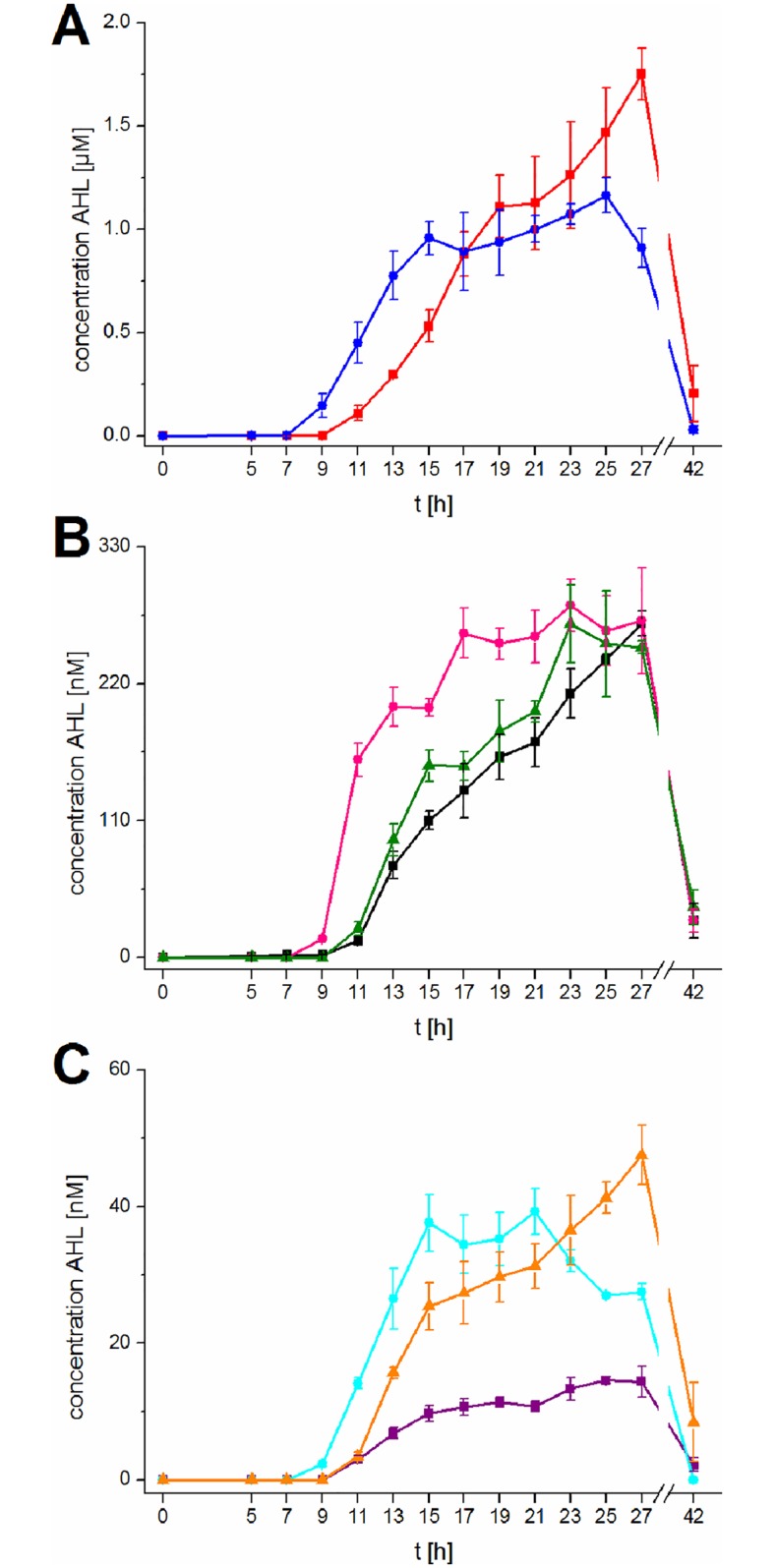
Production of AHLs in PB-St2; quantified using LC-MS/MS precursor ion scans and calibration curves. (A) 3-OH-C6-HSL (red, quantification was possible between 5 and 42 h) and 3-oxo-C6-HSL (blue, quantification was possible between 5 and 42 h). (B) C4-HSL (black, quantification was possible between 5 and 42 h), C6-HSL (pink, quantification was possible between 9 and 42 h), and 3-OH-C8-HSL (green, quantification was possible between 11 and 42 h). (C) C8-HSL (purple, quantification was possible between 11 and 42 h), 3-oxo-C8-HSL (cyan, quantification was possible between 9 and 27 h), and 3-OH-C10-HSL (orange, quantification was possible between 11 and 42 h). Data represent means with corresponding standard deviation of three independent replicates. When the amount was lower than the limit of quantification (see S2 Table) the production was considered as zero.

Production of 3-oxo-C6-HSL, C-6-HSL, and 3-oxo-C8-HSL started after 9 h of growth, shortly after the onset of the exponential phase, while the production of the remaining five AHLs started two hours later, after 11 hours in the middle of the exponential phase. Subsequently, the amount of all AHLs increased continuously, reaching a maximum after 15 hours for 3-oxo-C6-HSL, 3-oxo-C8-HSL, and C8-HSL at the end of the exponential phase. Maximum production of C6-HSL and 3-OH-C8-HSL was reached after 17 and 23 hours when the growth slowed down and cells were approaching the stationary phase. 3-OH-C6-HSL, C4-HSL, and 3-OH-C10-HSL did not reach to the maximum level until 27 hours, rather the production decreased after 19, 21, and 15 hours, respectively. 3-OH-C6-HSL was the predominantly produced AHL with a maximum concentration of 1.75±0.12 μM after 27 hours of growth. After passing through the maximum, the concentration of each AHL declined. At 42 hours growth, the amount of the AHLs was almost as low as at the beginning of production (Fig 4). Consequently, all AHLs were degraded during the stationary phase. The decline of 3-oxo-C6-HSL, 3-oxo-C8-HSL, and 3-OH-C-8-HSL started 27, 21, and 25 hours, respectively, after inoculation. To exclude the possibility that degradation occurs due to the increasing pH value during the fermentation process, the pH at every time point was measured. It was confirmed that the added 100 mM phosphate buffer keeps the pH stable between 6.3 and 7.1, for 42 hours (S5 Table). Thus, a BLASTp search was performed to identify AHL degrading enzymes in *P*. *aurantiaca* PB-St2. It revealed homologous genes with 67% and 58% protein sequence identity to the two AHL acylases QuiP and PvdQ from *P*. *aeruginosa* PAO1 [53, 54]. Furthermore, a homologue of the AHL acylase HacB from *P*. *syringae* pv. syringae B728a (71% protein sequence identity) was identified [55].

## Discussion

The genome analysis of *P*. *aurantiaca* PB-St2 revealed four putative canonical AHL synthases. A combined literature and bioinformatics survey for QS circuits in pseudomonads revealed that they possess also at the maximum four autoinducer synthases (e.g. *P*. *aeruginosa* PAO1), but then these systems consist of a mixture of canonical AHL synthases and non-AHL synthases (Table 3). However, many pseudomonads rely exclusively on AHL-based autoinducers. Considering solely AHL-based synthases, it becomes apparent that PB-St2 is outstanding since it possesses four putative AHL synthases of which three are functional. To the best of our knowledge, and exemplified by Table 3, all other pseudomonads contain at maximum three putative AHL-based autoinducer systems, of which possibly only two are functional, since *hdtS* is often identified, but its function as AHL synthase can currently not reliably be predicted and needs to be investigated on a case by case basis.

**Table 3:** QS systems of in-depth investigated *Pseudomonas* strains.

Organism	QS systems (number of AHL producing QS systems)	Reference
*P*. *chlororaphis* subsp. *aurantiaca* PB-St2	PhzI/R, CsaI/R, AurI/R, HdtS^a^ (3)	This study
*P*. *chlororaphis* subsp. *aurantiaca* StFRB508	PhzI/R, AurI/R (2)	[45]
*P*. *chlororaphis* subsp. *aureofaciens* 30–84	PhzI/R, CsaI/R, HdtS^a^ (2)	[36]
*P*. *aeruginosa* PAO1	LasI/R, RhlI/R, PqsABCDH/R AmbBCDE (2)	[48, 56]
*P*. *aeruginosa* PUPa3	LasI/R, RhlI/R, AmbBCDE^a^ (2)	[39]
*P*. *putida* IsoF	PpuI/R (1)	[57]
*P*. *putida* PCL1445	PpuI/R (1)	[58]
*P*. *putida* WCS358	PpuI/R (1)	[59]
*P*. *putida* BW11M1	n.d.^a^^,^^b^	[60]
*P*. *syringae* pv. syringae B728a	AhlI/R, HdtS^a^ (1)	[61]
*P*. *syringae* pv. tomato DC3000	PsyI/R, HdtS^a^ (1)	[62]
*P*. *syringae* pv. phaseolicola 1448A	AhlI/R^a^, HdtS^a^	[62]
*P*. *syringae* pv. maculicola CFBP 10912–9	PsmI/R (1)	[63]
*P*. *fluorescens* 2P24	PcoI/R (1)	[64]
*P*. *fluorescens* 2–79	PhzI/R (1)	[52]
*P*. *fluorescens* BBc6R8	HdtS^a^	[65]
*P*. *fluorescens* F113	HdtS (1)	[37]
*P*. *corrugata* strain CFBP 5454	PcoI/R, HdtS^a^ (1)	[66]
*P*. *fuscovaginae* UPB0736	PfsI/R, PfvI/R (2)	[67]
*P*. *protegens* Pf-5	HdtS ^a^	[68]
*P*. CMR12a	PhzI/R, CmrI/R (2)	[38]

^a^ Identified using BLASTp search (In case of HdtS: Identity to HdtS from *P*. *fluorescence* F113 > 80%) or antiSMASH 3.0 software.

^b^ no QS based system detected.

It was anticipated that *P*. *aurantiaca* PB-St2 produces more AHLs than the initially reported C6-HSL [12]. Indeed, seven additional AHLs were detected in the crude extract (Table 2), but homologues of the AHL synthases of other pseudomonads lead to the production of ten different AHLs [36, 37, 45]. Thus, heterologous expression of the AHL synthases in *E*. *coli* allowed the correlation of each produced AHL metabolite with its AHL synthase.

The HdtS homologue of PB-St2, did not produce any AHL when introduced into *E*. *coli*. Since no typical AHLs that are known to be produced by HdtS [37] were detected in *P*. *aurantiaca* PB-St2 extracts, it is reasonable that the HdtS homologue was either non-functional or does not act as an AHL synthase in *P*. *aurantiaca* PB-St2, but rather as a lysophosphatidic acid (LPA) acyltransferase. Notably, HdtS is not a member of the LuxI and the LuxM family of AHL synthases and belongs to a putative third class that is closely related to the LPA acyltransferase family [37]. It is proven that HdtS of *P*. *fluorescens* F113 acts as a LPA as well [69]. To the best of our knowledge, there is no proof of the function of HdtS *in vivo*, neither as an AHL synthase nor as a LPA. HdtS homologues are present in other gram negative strains [70–72]. The genomes of several pseudomonads are containing HdtS homologues with over 80% sequence identity to HdtS from *P*. *fluorescens* F113 (Table 3). In most of these strains, the production of AHLs was extensively studied [21, 36, 61, 62, 66, 68, 73] and there is no hint that HdtS is producing AHLs. This supports the assumption that HdtS homologues in *Pseudomonas* strains are not necessarily involved in the production of AHLs.

In previous studies AurI from *P*. *chlororaphis* subsp. *aurantiaca* StFRB508 was shown to produce alkyl-AHLs. In *Pseudomonas aurantiaca* PB-St2 AurI was the only AHL synthase responsible for the production of 3-oxo-AHLS. One of them, 3-oxo-C6-HSL, was detected in the natural producer *P*. *aurantiaca* PB-St2 in large amounts. The sequence similarity of AurI from PB-St2 and AurI from StFRB508 is extremely high with only four different amino acids in the conserved domain of the *N*-acetyltransferase super family portion of AurI which covers the interval of amino acid 11 through 191. We tested if the four amino acids were responsible for the shifted substrate specificity of the synthases by heterologous expressing *aurI* from StFRB508. The AHLs produced by AurI from StFRB508 were exactly the same as produced by AurI from PB-St2. Thus, the 3-oxo-AHLs produced by StFRB508 were possibly overlooked as a less sensitive TLC-based analytic methods and no 3-oxo-AHL was used as standard in this study. These findings lead to the conclusion that the AHL synthase AurI is able to produce 3-oxo-AHLs as well as alkyl-AHLs.

In pseudomonads, to the best of our knowledge so far the AhlI synthase from *P*. *syringae* pv. syringae B728a [61], the LasI synthase from *P*. *aeruginosa* PAO1 [74], and the PpuI synthase from *P*. *putida* PCL1445 [58] were proven to direct the biosynthesis of 3-oxo-AHLs. However, AurI of PB-St2 showed only a moderate protein sequence identity with AhlI (56%) and a low protein sequence identity with LasI (24%) and PpuI (22%), respectively. Consequently, AurI represents a further AHL synthase in pseudomonads that is responsible for the production of 3-oxo-AHLs with only slight homology to the previously characterized AHL synthases. In previous studies homologues of *aurI* were identified in *Pseudomonas putida*, *Pseudomonas fluorescens*, and *Pseudomonas syringae* [45]. Thus, the ability to produce 3-oxo-AHLs appears to be wide spread in pseudomonads and different types of AHL synthases are involved.

Since the effect of AHLs is concentration dependent, we quantified the amount of AHLs produced by *P*. *aurantiaca* PB-St2. It was previously shown for *P*. *aeruginosa* PAO1, that different AHLs are produced at different time points, according to the activity of the corresponding AHL synthase [74], thus, we conducted the quantification in a time-resolved mode. Up to now there are several excellent studies quantifying AHL production. However, most of them are either limited to the quantification of the produced AHLs at one or two time points [17, 71, 74–76] or to the time-dependent quantification of a single AHL metabolite and to AHLs, produced by only one AHL synthase [48, 53–55, 77–81]. We quantified for the first time all detectable AHLs in parallel, time dependent and correlated the production of them with the corresponding AHL synthase.

PhzI, is known to synthesize 3-OH-C6-HSL as the relevant quormone that is recognized by the regulator PhzR, which in turn is positively controlling phenazine production [36]. In *P*. *aurantiaca* PB-St2, 3-OH-C6-HSL is the predominant AHL with maximum production after 27 hours. PhzI is the only AHL synthase that is able to produce short chain 3-OH-AHLs, consequently we compared the onset of 3-OH-C6- and 3-OH-C8-HSL production with the production of phenazines. Phenazines were first detected after 13 hours of growth, shortly after the production of both 3-OH-AHLs started (11 hours of growth). With increasing amounts of both AHLs, the phenazine production raises simultaneously; an observation, which is in line with the fact that the PhzIR system is controlling phenazine production [40].

The QS system CsaIR is known to be only marginally involved in phenazine production. Its primary function appears to be the regulation of exoprotease production together with PhzI/R and the regulation of cell surface proteins [73]. In the case of CsaI, all AHLs are also produced by one of the other AHL synthases. Considering that C4-HSL is detected in the extracts of the heterologous host expressing CsaI in a very high amount (Fig 2D) in comparison to the extract of the host expressing AurI, we hypothesize that C4-HSL is the dominant AHL produced by CsaI. This is supported by the fact that CsaI from *P*. *aureofaciens* 30–84 also produces C4-HSL predominantly when introduced into *E*. *coli*. Likewise, for 3-OH-C6- and 3-OH-C8-HSL, the production of C4-HSL starts after 11 hours of growth. Thus, the CsaIR system is probably active at the same time frame as the PhzIR system.

Mediated by the AurIR system, 3-oxo-C6-HSL is produced in high amounts and is with 1.16±0.08 μM after 25 hours the only AHL that is produced almost in the same high amount as 3-OH-C6-HSL. Up to now, the specific control function of the AurI system was not yet discovered. Mutation experiments in strain *P*. *chlororaphis* subsp. aurantiaca StFRB508 revealed that it did not affect phenazine production [45]. However, AurI in strain PB-St2 might have a further, non-QS related function due to the production of 3-oxo-AHLs. In this context it is noteworthy to mention that very recently, 3-oxo-C8-HSL was found to stimulate the growth of sugarcane at a micromolar scale, measured by an increased bud length, dry matter weight, fresh root mass, and dry root mass in comparison to the untreated control plants [82]. Since micromolar concentrations are sufficient for this effect, further studies will show if *P*. *aurantiaca* PB-St2 is not only protecting sugarcane from the red rot disease, but also promoting its growth through AHL production. Notably, AurI starts the biosynthesis of AHLs after nine hours, which is two hours earlier than CsaI and PhzI. In line with the early onset of production, the maximum amount is produced very soon after additional six hours and degradation began after another six hours. Thus, our study showed that the time point for analyzing AHLs and especially for their quantification is very important. For comparison of the amount of single AHLs species, several time points are needed because the maximum is not reached at the same time.

The time point for the detection of AHLs is of great importance as almost complete degradation was observed for all AHLs after 42 hours of growth. As the pH value did not exceed 7.10±0.01, it is unlikely that degradation occurred due to pH dependent lactonolysis, as described previously [83–85]. Most probably, degradation is caused by AHL acylase activity. Using BLASTp we identified homologues to the two AHL acylases QuiP and PvdQ from *P*. *aeruginosa* PAO1 [53, 54] and to the AHL acylase HacB from *P*. *syringae* pv. syringae B728a [55]. QuiP and PvdQ are known for the degradation of long chain (C ≥ 8) AHLs in *P*. *aeruginosa* PAO1, which would affect only half of the AHLs produced by *P*. *aurantiaca* PB-St2. Homologues for PvdQ (HacA), QuiP (Psyr_3871), and for PA0305 (HacB), a third uncharacterized protein from *P*. *aeruginosa*, were identified and analyzed in *P*. *syringae* pv. syringae B728a. In this strain, the QuiP homologue is not inactivating AHLs, while HacA is degrading C8-HSL, C10-HSL, and C12-HSL. HacB is degrading 3-oxo-C6-HSL, C6-HSL, C8-HSL, C10-HSL, and C12-HSL [55]. Thus, these three enzymes could be responsible for the observed degradation of AHLs after 42 hours of cultivation.

## Conclusions

In summary, we revealed that QS in *P*. *aurantiaca* PB-St2 is mediated by three active AHL based QS systems in one single microorganism. Thus, *P*. *aurantiaca* PB-St2 is until today to the best of our knowledge the first *Pseudomonas* strain that is using three functional AHL based QS systems in parallel (Table 3). We quantified the full AHL spectrum of PB-St2 and shed further light on the time-dependent production of its biosynthesized AHLs, correlated the AHL metabolites with their genetic origin, and revealed AurI as a new biosynthetic system that is able to produce 3-oxo-AHLs. Altogether, our study helps to understand QS in *P*. *aurantiaca* PB-St2, which is more complex as previously expected. Further studies will focus on determining how the three QS systems interact with each other and their effect on rhizosphere colonization and biocontrol activity.

## Supporting Information

S1 Fig Genomic vicinity of the putative QS system genes. Genes marked in red: *phzI/R* (A), *csaI/R* (B), *aurI/R* (C), and *hdtS* (D). Genes are labeled with the putative encoded enzyme. Genes coding for hypothetical proteins are not labeled. Regulatory genes other than QS related, lipid biosynthesis genes, and phenazine biosynthesis genes are indicated in yellow, purple, and green, respectively. ACP = acyl carrier protein, DH = dehydrogenase, Gsp = general secretion pathway, Asn = aconitate hydratase.
(TIF)

S2 Fig Identification of AHLs produced by *P*. *aurantiaca* PB-St2 using precursor ion scan. Extracted ion chromatograms (LC-MS/MS, precursor ion scan, positive ionization mode) of *P*. *aurantiaca* PB-St2 extracts (black), the corresponding standard AHLs (red), and 1:1 mixtures of *P*. *aurantiaca* PB-St2 extract and standard AHL (green). Applied standard AHL and corresponding extracted ions: (A) C4-HSL (*m/z* 172–173), (B) C6-HSL (*m/z* 200–201), (C) C8-HSL (*m/z* 228–229), (D) 3-OH-C6-HSL (*m/z* 216–217), (E) 3-OH-C8-HSL (*m/z* 244–245), (F) 3-OH-C10-HSL (*m/z* 272–273), (G) 3-oxo-C6-HSL (*m/z* 214–215), and (H) 3-oxo-C8-HSL (*m/z* 242–243).
(TIF)

S3 Fig Identification of AHLs produced by *P*. *aurantiaca* PB-St2 using product ion scan. Total ion chromatograms (LC-MS/MS, product ion scan, positive ionization mode) of *P*. *aurantiaca* PB-St2 extracts (black), the corresponding standard AHLs (red), and 1:1 mixtures of *P*. *aurantiaca* PB-St2 extract and standard AHL (green). Applied standard AHL and corresponding fragmented ions: (A) C4-HSL (*m/z* 172.2), (B) C6-HSL (*m/z* 200.4), (C) C8-HSL (*m/z* 228.2), (D) 3-OH-C6-HSL (*m/z* 216.2), (E) 3-OH-C8-HSL (*m/z* 244.2), (F) 3-OH-C10-HSL (*m/z* 272.2), (G) 3-oxo-C6-HSL (*m/z* 214.1), and (H) 3-oxo-C8-HSL (*m/z* 242.2).
(TIF)

S4 Fig Comparison of LC-MS/MS spectra to identify AHLs produced by *P*. *aurantiaca* PB-St2. LC-MS/MS spectra (product ion scan, positive ionization mode) of *P*. *aurantiaca* PB-St2 extracts (black), the corresponding standard AHLs (red), and 1:1 mixtures of *P*. *aurantiaca* PB-St2 extract and standard AHL (green). Applied standard AHL (corresponding fragmented ions, time the spectrum was extracted): (A) C4-HSL (*m/z* 172.2, 12.5 min), (B) C6-HSL (*m/z* 200.4, 19.9 min), (C) C8-HSL (*m/z* 228.2, 24.9 min), (D) 3-OH-C6-HSL (*m/z* 216.2, 14.2 min), (E) 3-OH-C8-HSL (*m/z* 244.2, 20.0 min), (F) 3-OH-C10-HSL (*m/z* 272.2, 24.2 min), (G) 3-oxo-C6-HSL (*m/z* 214.1, 15.3 min), and (H) 3-oxo-C8-HSL (*m/z* 242.2, 21.5 min).
(TIF)

S5 Fig C6-HSL is produced by HdtS from *P*. *fluorescens* F113, but not by HdtS from *P*. *aurantiaca* PB-St2. Extracted ion chromatograms (LC-MS/MS, precursor ion scan, positive ionization mode) of C6-HSL produced by *E*. *coli* XL1-Blue expressing either *hdtS* of *P*. *aurantiaca* PB-St2 (black) or *hdtS* of *P*. *fluorescens* F113 (red). Extracted ions: *m/z* 200–201.
(TIF)

S6 Fig AurI from *P*. *aurantiaca* PB-St2 and AurI from *P*. *chlororaphis* subsp. *aurantiaca* StFRB508 are producing the same AHLs. Extracted ion chromatograms (LC-MS/MS, precursor ion scan, positive ionization mode) of [M+H]^+^ ions of AHLs present in extracts of *E*. *coli* XL1-Blue expressing (A) *aurI* from *P*. *aurantiaca* PB-St2 and (B) *aurI* from *P*. *chlororaphis* subsp. *aurantiaca* StFRB508. C4-HSL (black, *m/z* 172–173), 3-oxo-C6-HSL (blue, *m/z* 214–215), C6-HSL (pink, *m/z* 200–201), 3-oxo-C8-HSL (cyan, *m/z* 242–243), C8-HSL (purple, *m/z* 228–229).
(TIF)

S7 Fig C9-HSL is a suitable standard for the quantification of AHLs in *P*. *aurantiaca* PB-St2. Extracted ion chromatograms (LC-MS/MS, precursor ion scan, positive ionization mode) of *P*. *aurantiaca* PB-St2 extract (black), C9-HSL standard (red), and 1:1 mixture of *P*. *aurantiaca* PB-St2 extract and C9-HSL (green). Extracted ions: *m/z* 242–243.
(TIF)

S8 Fig Calibration curves for AHL quantification. Data represent means with corresponding standard deviation of three independent replicates. Red lines show the linear fit.
(TIF)

S1 Table Nucleotide Sequences of putative AHL Synthases from *P*. *aurantiaca* PB-St2. (DOCX)

S2 Table Nucleotide Sequence of *hdtS* from *P*. *fluorescens* F113. (DOCX)

S3 Table Nucleotide Sequence of *aurI* from *P*. *chlororaphis* subsp. *aurantiaca* StFRB508. (DOCX)

S4 Table Calibration equation and limit of quantification used for quantification of the corresponding AHLs. (DOCX)

S5 Table Measured pH values of *P*. *aurantiaca* PB-St2 culture supernatants at different growth intervals. (DOCX)

S6 Table Sequence alignment of AurI. (DOCX)
