# Supplementary figures and images for: The Systematic Investigation of the Quorum Sensing System of the Biocontrol Strain Pseudomonas chlororaphis subsp. aurantiaca PB-St2 Unveils aurI to Be a Biosynthetic Origin for 3-Oxo-Homoserine Lactones
Source: PLoS One. 2016 Nov 18;11(11):e0167002. doi: 10.1371/journal.pone.0167002 (PMC5115851; doi:10.1371/journal.pone.0167002)

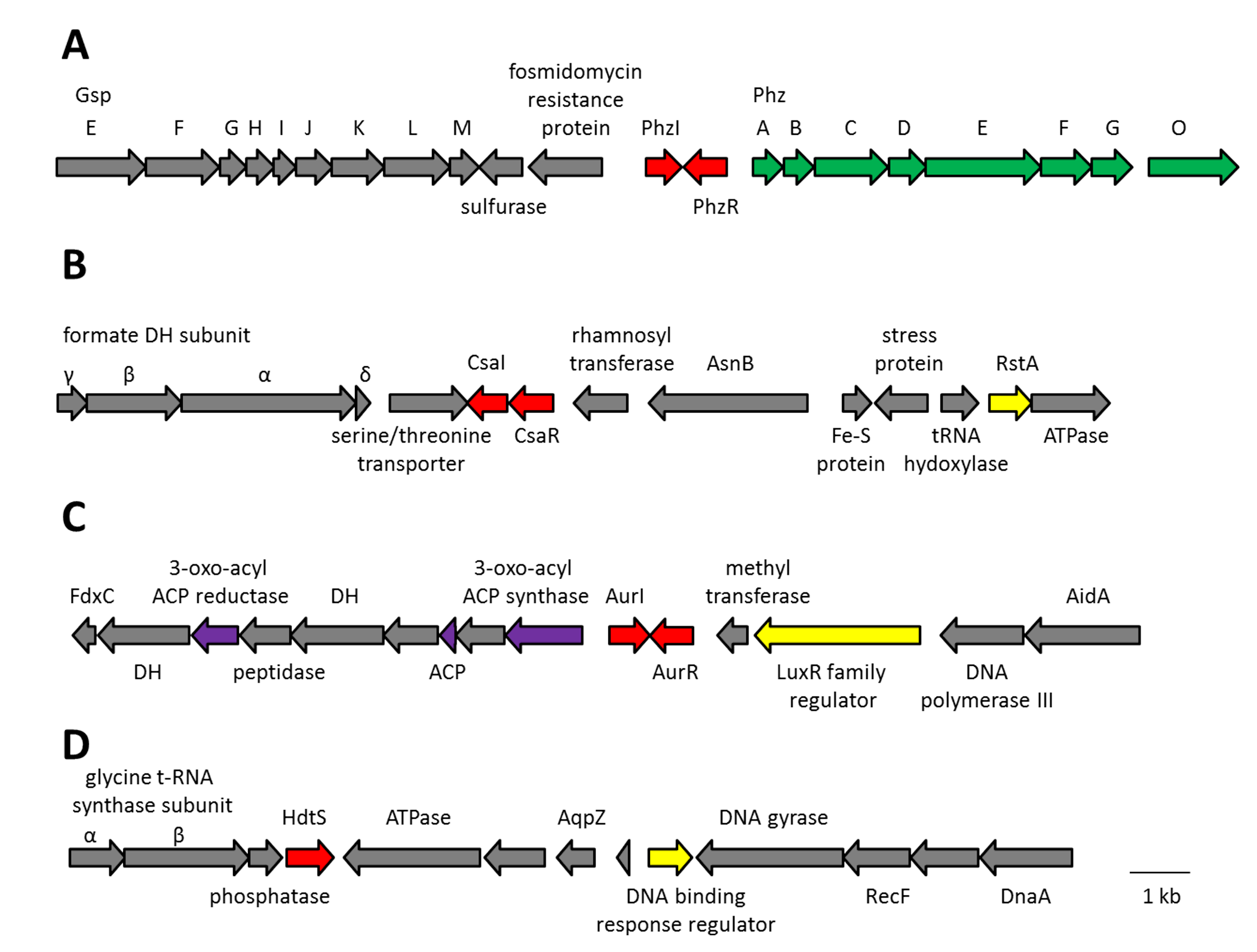

Supplement: S1 Fig — Genes marked in red: phzI/R (A), csaI/R (B), aurI/R (C), and hdtS (D). Genes are labeled with the putative encoded enzyme. Genes coding for hypothetical proteins are not labeled. Regulatory genes other than QS related, lipid biosynthesis genes, and phenazine biosynthesis genes are indicated in yellow, purple, and green, respectively. ACP = acyl carrier protein, DH = dehydrogenase, Gsp = general secretion pathway, Asn = aconitate hydratase. (TIF) [file pone.0167002.s001.tif]

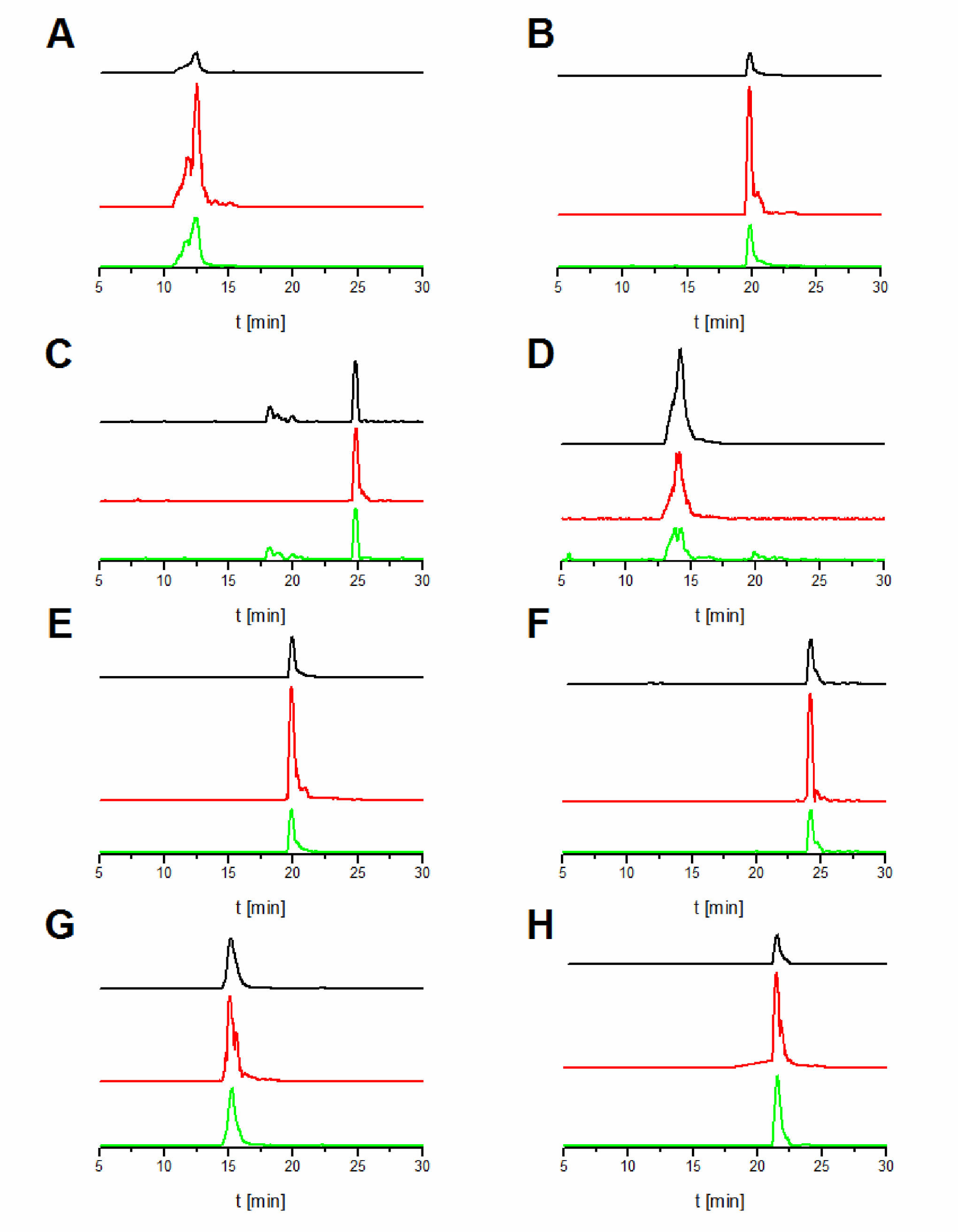

Supplement: S2 Fig — Extracted ion chromatograms (LC-MS/MS, precursor ion scan, positive ionization mode) of P. aurantiaca PB-St2 extracts (black), the corresponding standard AHLs (red), and 1:1 mixtures of P. aurantiaca PB-St2 extract and standard AHL (green). Applied standard AHL and corresponding extracted ions: (A) C4-HSL (m/z 172–173), (B) C6-HSL (m/z 200–201), (C) C8-HSL (m/z 228–229), (D) 3-OH-C6-HSL (m/z 216–217), (E) 3-OH-C8-HSL (m/z 244–245), (F) 3-OH-C10-HSL (m/z 272–273), (G) 3-oxo-C6-HSL (m/z 214–215), and (H) 3-oxo-C8-HSL (m/z 242–243). (TIF) [file pone.0167002.s002.tif]

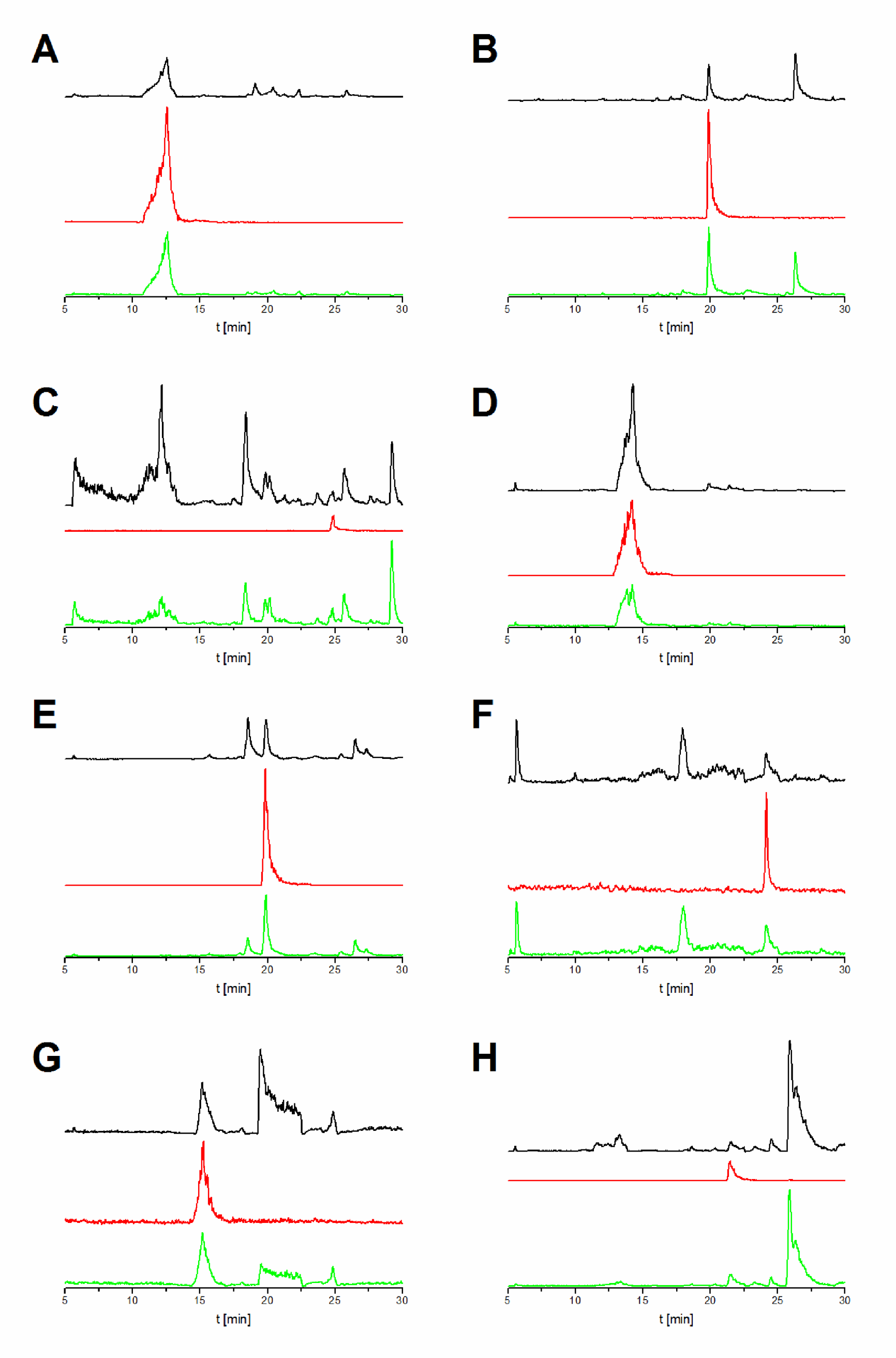

Supplement: S3 Fig — Total ion chromatograms (LC-MS/MS, product ion scan, positive ionization mode) of P. aurantiaca PB-St2 extracts (black), the corresponding standard AHLs (red), and 1:1 mixtures of P. aurantiaca PB-St2 extract and standard AHL (green). Applied standard AHL and corresponding fragmented ions: (A) C4-HSL (m/z 172.2), (B) C6-HSL (m/z 200.4), (C) C8-HSL (m/z 228.2), (D) 3-OH-C6-HSL (m/z 216.2), (E) 3-OH-C8-HSL (m/z 244.2), (F) 3-OH-C10-HSL (m/z 272.2), (G) 3-oxo-C6-HSL (m/z 214.1), and (H) 3-oxo-C8-HSL (m/z 242.2). (TIF) [file pone.0167002.s003.tif]

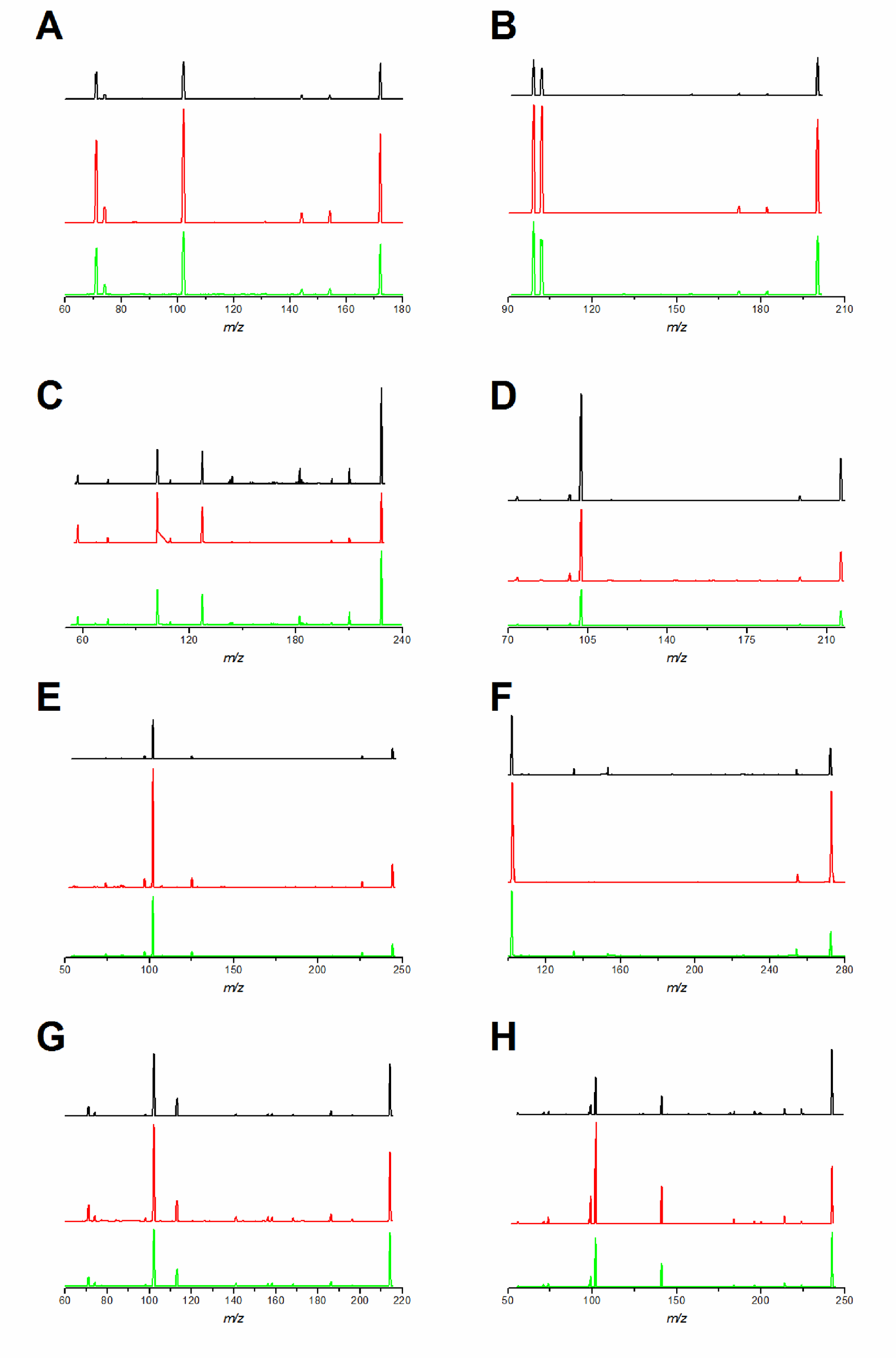

Supplement: S4 Fig — LC-MS/MS spectra (product ion scan, positive ionization mode) of P. aurantiaca PB-St2 extracts (black), the corresponding standard AHLs (red), and 1:1 mixtures of P. aurantiaca PB-St2 extract and standard AHL (green). Applied standard AHL (corresponding fragmented ions, time the spectrum was extracted): (A) C4-HSL (m/z 172.2, 12.5 min), (B) C6-HSL (m/z 200.4, 19.9 min), (C) C8-HSL (m/z 228.2, 24.9 min), (D) 3-OH-C6-HSL (m/z 216.2, 14.2 min), (E) 3-OH-C8-HSL (m/z 244.2, 20.0 min), (F) 3-OH-C10-HSL (m/z 272.2, 24.2 min), (G) 3-oxo-C6-HSL (m/z 214.1, 15.3 min), and (H) 3-oxo-C8-HSL (m/z 242.2, 21.5 min). (TIF) [file pone.0167002.s004.tif]

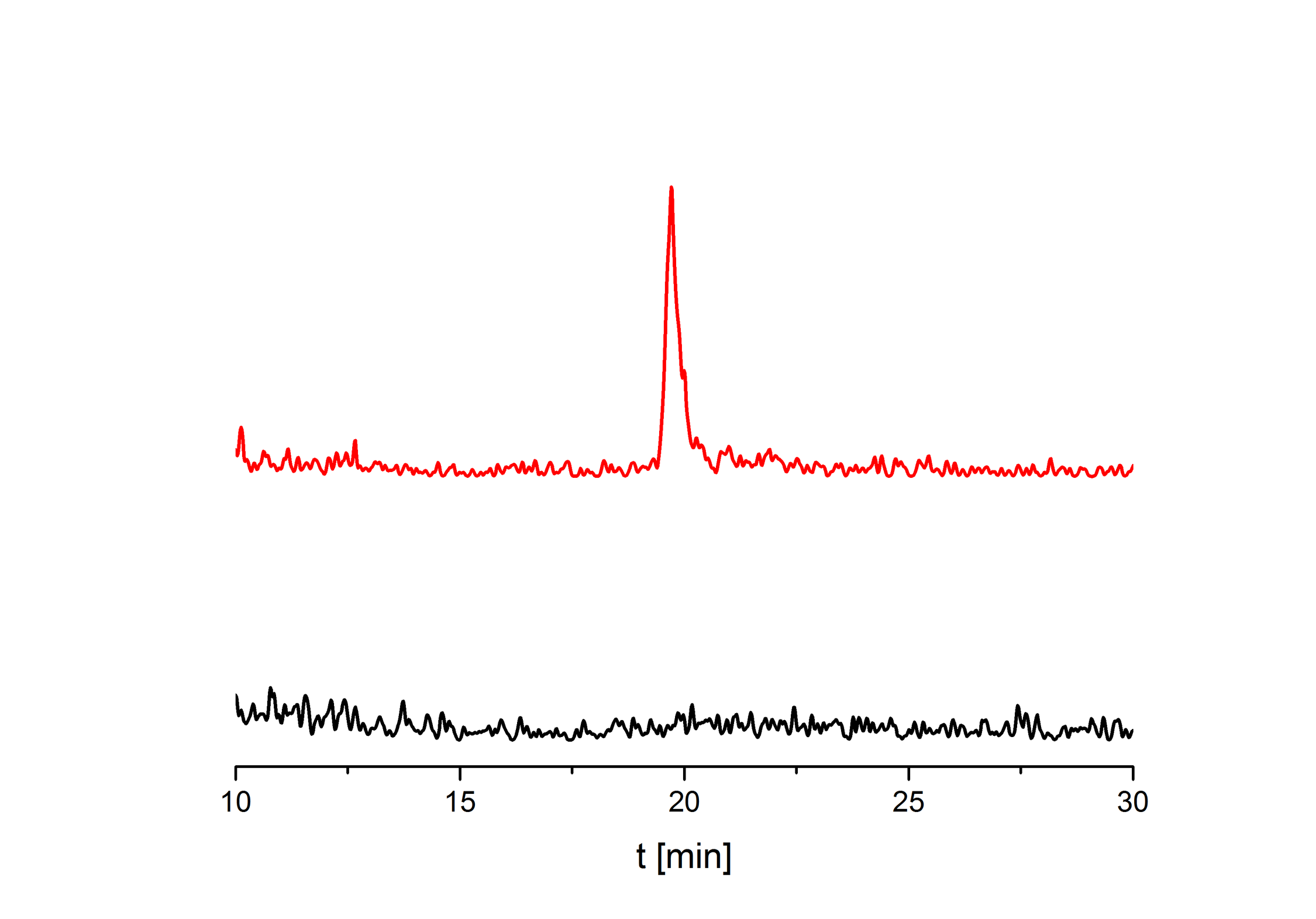

Supplement: S5 Fig — Extracted ion chromatograms (LC-MS/MS, precursor ion scan, positive ionization mode) of C6-HSL produced by E. coli XL1-Blue expressing either hdtS of P. aurantiaca PB-St2 (black) or hdtS of P. fluorescens F113 (red). Extracted ions: m/z 200–201. (TIF) [file pone.0167002.s005.tif]

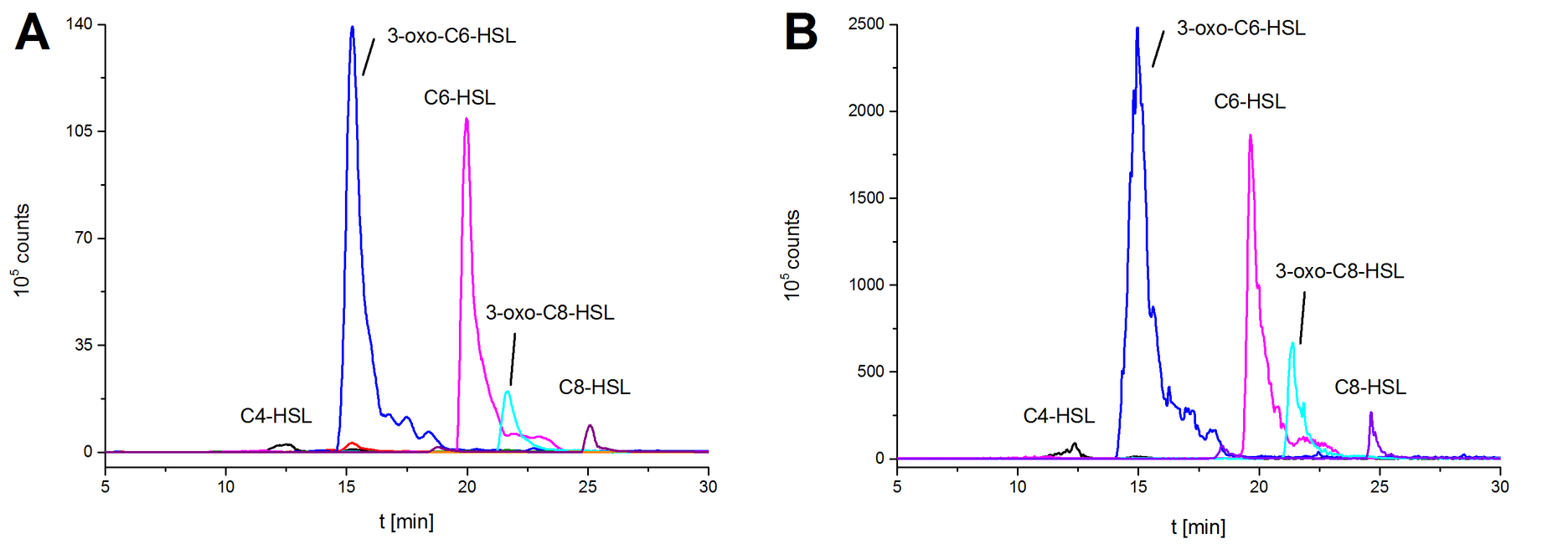

Supplement: S6 Fig — Extracted ion chromatograms (LC-MS/MS, precursor ion scan, positive ionization mode) of [M+H]+ ions of AHLs present in extracts of E. coli XL1-Blue expressing (A) aurI from P. aurantiaca PB-St2 and (B) aurI from P. chlororaphis subsp. aurantiaca StFRB508. C4-HSL (black, m/z 172–173), 3-oxo-C6-HSL (blue, m/z 214–215), C6-HSL (pink, m/z 200–201), 3-oxo-C8-HSL (cyan, m/z 242–243), C8-HSL (purple, m/z 228–229). (TIF) [file pone.0167002.s006.tif]

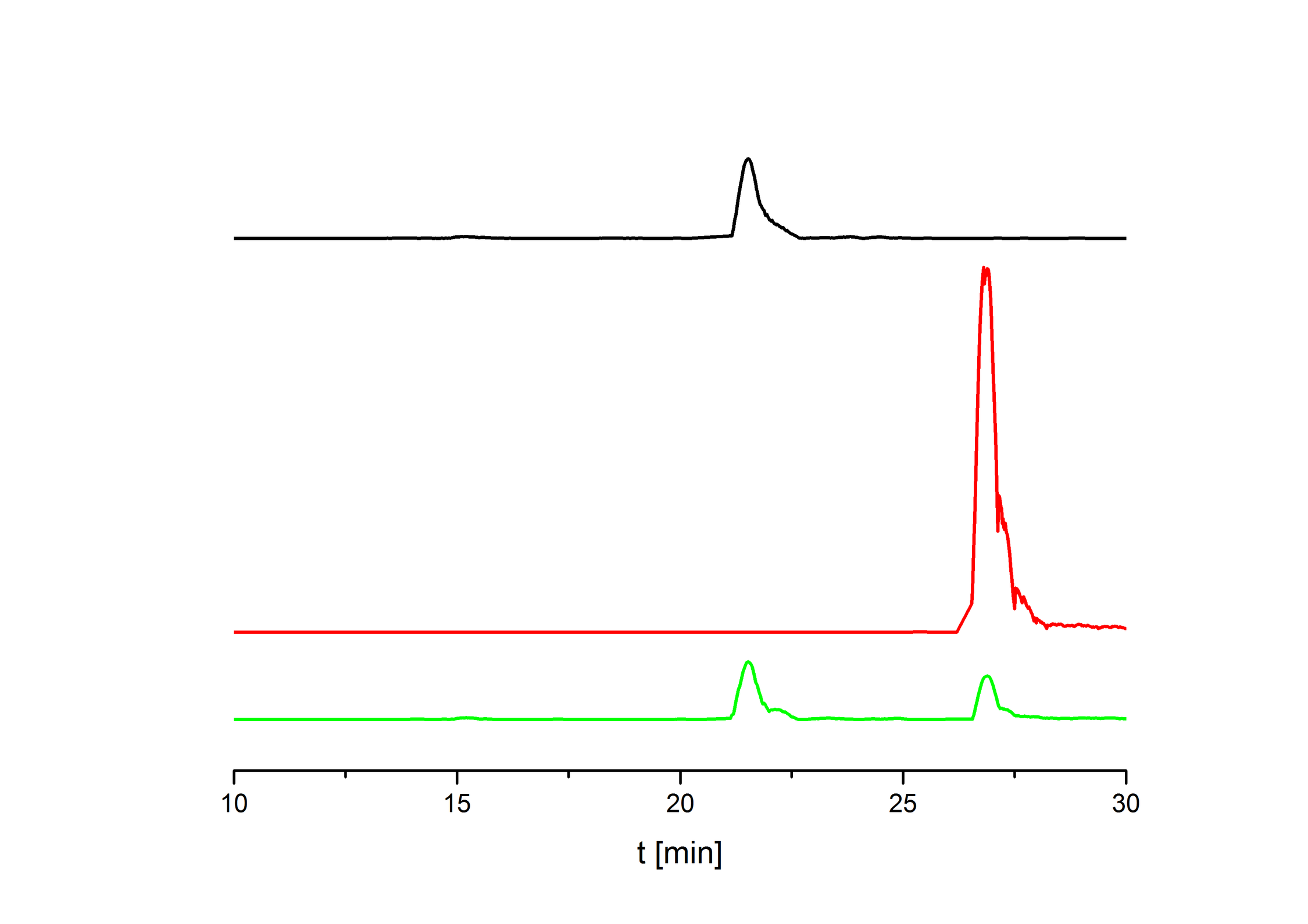

Supplement: S7 Fig — Extracted ion chromatograms (LC-MS/MS, precursor ion scan, positive ionization mode) of P. aurantiaca PB-St2 extract (black), C9-HSL standard (red), and 1:1 mixture of P. aurantiaca PB-St2 extract and C9-HSL (green). Extracted ions: m/z 242–243. (TIF) [file pone.0167002.s007.tif]

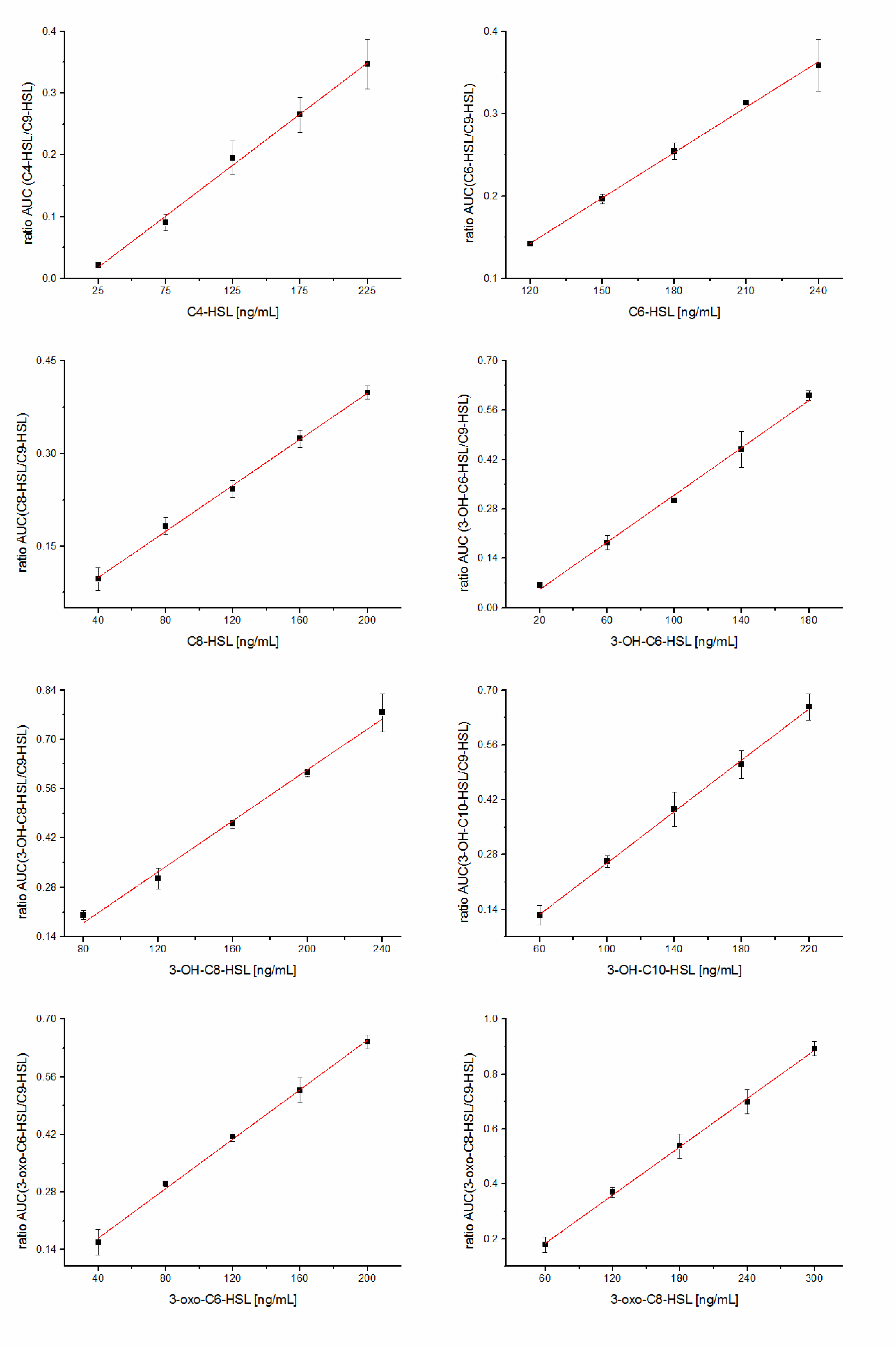

Supplement: S8 Fig — Data represent means with corresponding standard deviation of three independent replicates. Red lines show the linear fit. (TIF) [file pone.0167002.s008.tif]
