# Supplementary material for: The Systematic Investigation of the Quorum Sensing System of the Biocontrol Strain Pseudomonas chlororaphis subsp. aurantiaca PB-St2 Unveils aurI to Be a Biosynthetic Origin for 3-Oxo-Homoserine Lactones
Source: PLoS One. 2016 Nov 18;11(11):e0167002. doi: 10.1371/journal.pone.0167002 (PMC5115851; doi:10.1371/journal.pone.0167002)
Supplement: S1 Table — (DOCX) [file pone.0167002.s009.docx]

**S1 Table. Nucleotide Sequences of putative AHL Synthases from *P. aurantiaca* PB-St2.**

| *aurI*_ PciI*_lac*-promotor: |
| --- |
| 1 AAAAAAACAT GTTCTTTCCT GCGTTATCCC CTGATTCTGT GGATAACCGT ATTACCGCCT TTGAGTGAGC TGATACCGCT  81 CGCCGCAGCC GAACGACCGA GCGCAGCGAG TCAGTGAGCG AGGAAGCGGA AGAGCGCCCA ATACGCAAAC CGCCTCTCCC  161 CGCGCGTTGG CCGATTCATT AATGCAGCTG GCACGACAGG TTTCCCGACT GGAAAGCGGG CAGTGAGCGC AACGCAATTA  241 ATGTGAGTTA GCTCACTCAT TAGGCACCCC AGGCTTTACA CTTTATGCTT CCGGCTCGTA TGTTGTGTGG AATTGTGAGC  *lac*-promotor *lac*-operator  321 GGATAACAAT TTCACACAGG AAACAGCTAT GGAATCCATC GAATTTCACG CGCTTGACTA TAGTGCGACG CCCCACGCCT  *aur*I  401 GGGTCGCCGA TCTGCATGGC CTGCGCAAGG AAGTATTCGC CGATCGTTTG AACTGGAAGG TTAATATAAA GAATGACATC  481 GAGTTCGATG AGTACGACAA CGAGCGCACC ACCTACCTGA TCGGTACCTG GAAAGGTGTG CCTCTGGCCG GCCTGCGCCT  561 GATCAACACC CTGGATCCCT ACATGGTCGA AGGGCCGTTC CGCGACTTTT TCCGCTGCGA GCCGCCCAAG CAGGCGTTGA  641 TGGCTGAATC CAGCCGCTTT TTCGTCGACA AGACCCGCTC GCGCCAGCTC GGCCTGGCCC ATCTGCCGCT GACCGAAATG  721 CTCCTGTTGT GTATGCACAA CCATGCCGCG CGCAGCGGCC TGGAATCGAT CATCACGGTG GTCAGCAGCG CCATGGGGCG  801 GATCGTCCGC AACGCCGGCT GGCACTACGA AGTAATGGAC ACCGGCGAGG CCGCGCCGGG AGAAAAGGTG CTGTTGCTGA  881 ACATGCCGAT CAGCGACGCC AATCGTCAGC GCCTGCTGTC CAGCATCGCC CGCAAATGCC CCTTGTCATC CGCGCAGCTC  961 AACCATTGGC CGCAGCGCCT GAACCCGCTC CACACCGCGC TCTGCGAGCC TCAACGGAT**T** AGCGC**A**TGA |
| *csaI*_PciI_*lac*-promotor: |
| 1 AAAAAAACAT GTTCTTTCCT GCGTTATCCC CTGATTCTGT GGATAACCGT ATTACCGCCT TTGAGTGAGC TGATACCGCT  81 CGCCGCAGCC GAACGACCGA GCGCAGCGAG TCAGTGAGCG AGGAAGCGGA AGAGCGCCCA ATACGCAAAC CGCCTCTCCC  161 CGCGCGTTGG CCGATTCATT AATGCAGCTG GCACGACAGG TTTCCCGACT GGAAAGCGGG CAGTGAGCGC AACGCAATTA  241 ATGTGAGTTA GCTCACTCAT TAGGCACCCC AGGCTTTACA CTTTATGCTT CCGGCTCGTA TGTTGTGTGG AATTGTGAGC  *lac*-promotor *lac*-operator  321 GGATAACAAT TTCACACAGG AAACAGCTAT GATCACTGTG ATTTCACGGC ATGAAAGCCA GCTTTCACCG ACACTGCGCG  *csa*I  401 ACGACCTCGG CCGCTATCGC CATGCGGTCT TCATCAAGCA ACTGGGCTGG CGACTGCCCG CCGGCACCCG CCAGTGCGGG  481 CACGAGGTCG ATCAGTTCGA CCATGCCGAC ACCCGCTACA CCCTGGCGCT GGACAGCGAG GACAAGATCC ACGGCTGCGC  561 CCGCTTGCTG CCGACCACCC AGCCCTATCT GCTGGCGGAC GTGTTCGGTT TCCTCTGCGA CCGCCCCTTG CCGCGGCAGC  641 ACGACACCTG GGAAATCTCG CGTTTCGCCG CCTCGGCCCT GGAAAACGGC AAGCTGCCGA TGCGGGTGTT CTGGCACACC  721 CTGCACACCG CCTGGACCCT GGGCGCGAAC TCGGTGGTGG CGGTGACCAC GCCAGCGCTG GAGCGTTATT TCCTGCGCCA  801 TGGCGTGGCG CTGAGCCGGC TCGGCCAGCC GCAACGGGTC AACCGCGACC ACTTGCTGGC CCTGGACTTT CCGGCCTACC  881 AGAAAAACGG CCGCGCCGCG CTTTATACGC AGTCAGCGGC CGTGGCTTCG CTGAATCAGG CATTTCTGCG CGGCAACCCA  961 CCGCCAGCAC GCGGTGGGCC GCCGGCGGGT CAGGCGCTCA GGGAGTAG |
| *hdtS*_PciI_*lac*-promotor: |
| 1 AAAAAAACAT GTTCTTTCCT GCGTTATCCC CTGATTCTGT GGATAACCGT ATTACCGCCT TTGAGTGAGC TGATACCGCT  81 CGCCGCAGCC GAACGACCGA GCGCAGCGAG TCAGTGAGCG AGGAAGCGGA AGAGCGCCCA ATACGCAAAC CGCCTCTCCC  161 CGCGCGTTGG CCGATTCATT AATGCAGCTG GCACGACAGG TTTCCCGACT GGAAAGCGGG CAGTGAGCGC AACGCAATTA  241 ATGTGAGTTA GCTCACTCAT TAGGCACCCC AGGCTTTACA CTTTATGCTT CCGGCTCGTA TGTTGTGTGG AATTGTGAGC  *lac*-promotor *lac*-operator  321 GGATAACAAT TTCACACAGG AAACAGCTAT GTCGATCCTG CAGGCAATCA GAACCTTCTT CTTTTACCTG CTGCTGGGCA  *hdtS*  401 CCAGTTCGTT GCTGTGGTGC TCCCTGAGCT TTTTTATCGC GCCTTTCCTG CCGTTCAAGG CGCGCTATCG CTTTATCAAC  481 GTCTATTGGT GCCGCTGCGC GTTGTGGCTG GCCAAGGTGT TTCTCAAGAT CAACGTGGAA GTGAAGGGCG CGGAAAACGT  561 CCCCGAGCGT CCTTGCGTGA TTGTCTCGAA CCACCAGAGC ACCTGGGAGA CGTTCTTTCT CTCGGCCTAT TTCGAACCGT  641 TGAGCCAGGT GCTCAAGCGT GAACTGCTGT ACGTGCCGTT CTTCGGCTGG GCCATGGCCA TGCTGCGTCC GATCGCCATC  721 GACCGCGACA ACCCCAAGGC CGCGCTCAAG CAGGTCGCCA GCAAGGGCGA CGAGTTGCTC AAGGACAATG TCTGGGTACT  801 GATCTTCCCG GAAGGCACAC GGGTGCCTTA CGGCCAGATG GGCAAGTTCT CCCGCAGCGG CAGCGCCCTG GCGGTCAACG  881 CCGACTTGCC GGTGCTGCCG GTGGCGCACA ATGCCGGCAA ATACTGGCCG AAGACCGGCT GGGTCAGGCA CCCTGGCACC  961 ATCACCGTGG TGATCGGTGC GCCGATGTAC GCCGAAGGCT CCGGACCACG GGCCATCGCC GAGCTCAACG ACCGGGTCGC  1041 GGCCTGGAAT GAGCAGGCGC AGCGGGACAT GGGTTCGCTG CCTCCGGTAG CGGCGGCACC GGACAAGATG GCCATCTGA |
| *phzI*_PciI_*lac*-promotor: |
| 1 AAAAAAACAT GTTCTTTCCT GCGTTATCCC CTGATTCTGT GGATAACCGT ATTACCGCCT TTGAGTGAGC TGATACCGCT  81 CGCCGCAGCC GAACGACCGA GCGCAGCGAG TCAGTGAGCG AGGAAGCGGA AGAGCGCCCA ATACGCAAAC CGCCTCTCCC  161 CGCGCGTTGG CCGATTCATT AATGCAGCTG GCACGACAGG TTTCCCGACT GGAAAGCGGG CAGTGAGCGC AACGCAATTA  241 ATGTGAGTTA GCTCACTCAT TAGGCACCCC AGGCTTTACA CTTTATGCTT CCGGCTCGTA TGTTGTGTGG AATTGTGAGC  *lac*-promotor *lac*-operator  321 GGATAACAAT TTCACACAGG AAACAGCTAT GCACATGGAA GAGCACACAC TGAACGAAAT GAGCGATGAG CTGAAACTCA  *phz*I  401 TGCTCGGCCG TTTTCGGCAC GAACAATTCG TCGAGAAACT CGGATGGCGA CTGCCCGCCC ACCCGAGCCA GGCAGGTTGT  481 GAATGGGACC GATACGACAC CGAACACGCC CGTTACCTCC TGGCGTTCAA TGCAGACCGC GCCATCGTTG GCTGCGCCCG  561 GCTGATTCCC ACCACGCTCC CCAACCTGCT TGAAGGGGTG TTCAGCCATG CCTGTGCCGG GACGCCGCCC AAGCATCCAG  641 CCATCTGGGA AATGACTCGC TTCACCACCT GCGAACCGCA ATTGGCGATG CCGTTGTTCT GGAGAAGCCT CAAGACGGCC  721 GCCCAGGCGG GCGCAGAGGC CATTGTCGGG ATCGTCAACA GCACCATGGA GCGCTATTAC AAAATCAATG GCGTCCACTA  801 CGAACGGCTG GGCCCGGTCA CGGTGCACCA GAATGAGAAA ATCCTCGCCA TCAAACTCTC GGCCCACCGC GAGCACCATC  881 GCAGCGCGGT CGCACCGTCA GCCTTCATGT CCGGCACATT ATTGAAAGAG ACAGCTTGA |
|  |
|  |
