# Supplementary material for: The Systematic Investigation of the Quorum Sensing System of the Biocontrol Strain Pseudomonas chlororaphis subsp. aurantiaca PB-St2 Unveils aurI to Be a Biosynthetic Origin for 3-Oxo-Homoserine Lactones
Source: PLoS One. 2016 Nov 18;11(11):e0167002. doi: 10.1371/journal.pone.0167002 (PMC5115851; doi:10.1371/journal.pone.0167002)
Supplement: S2 Table — (DOCX) [file pone.0167002.s010.docx]

**S2 Table. Nucleotide Sequence of *hdtS* from *P. fluorescens* F113.**

| *hdtS*_F113_PciI_*lac*-promotor: |
| --- |
| 1 AAAAAAACAT GTTCTTTCCT GCGTTATCCC CTGATTCTGT GGATAACCGT ATTACCGCCT TTGAGTGAGC TGATACCGCT  81 CGCCGCAGCC GAACGACCGA GCGCAGCGAG TCAGTGAGCG AGGAAGCGGA AGAGCGCCCA ATACGCAAAC CGCCTCTCCC  161 CGCGCGTTGG CCGATTCATT AATGCAGCTG GCACGACAGG TTTCCCGACT GGAAAGCGGG CAGTGAGCGC AACGCAATTA  241 ATGTGAGTTA GCTCACTCAT TAGGCACCCC AGGCTTTACA CTTTATGCTT CCGGCTCGTA TGTTGTGTGG AATTGTGAGC  >> *lac*-promotor >> >> *lac*-  321 GGATAACAAT TTCACACAGG AAACAGCTAT GTCGATATTG CAGGCCATCA GAGCCTTTCT CTTTTACCTG CTGTTGGGCA  operator >> >> *hdtS*  401 CCACTTCGTT GCTGTGGTGC TCCTTGAGTT TTTTTATCGC GCCTTTCTTG CCGTTCAAGG CGCGCTACCG TTTCATCAAT  481 GTGTACTGGT GCCACTGCGC ACTGTGGTTG AGCAAGGTGT TCCTGGGCAT CCGCTATGAA GTCAAAGGTG CCGAGAACGT  561 GCCCGACCGG CCCTGCGTGA TCCAGTCCAA CCACCAGAGC ACCTGGGAGA CGTTCTTTCT CTCGGCCTAT TTCGAACCGT  641 TGAGCCAAGT GCTCAAGCGT GAACTGTTGT TCGTGCCGTT CTTCGGCTGG GCCATGGCGA TGCTGCGCCC GATCGCCATC  721 GATCGCGACA ACCCCAAGGC GGCCCTCAAG CAGGTCGCGA AGAAGGGTGA CGAACTGCTC AAGGATAACG TTTGGGTGCT  801 GATCTTCCCC GAGGGCACCC GTGTTCCTTA TGGAACCGTC GGCAAATTCT CCCGCAGCGG TTCGGCATTG GCCGTGAACG  881 CCGATCTTCC CGTGCTGCCG ATTGCACACA ATGCCGGCAA ATTCTGGCCC AAGGCTGGCT GGATCAGGAA ACCAGGCGTC  961 ATCACTGTTG TGATCGGCGC GCCGATGTAT GCCGAAGGCA CTGGGCCCCG CGCCATCGCC GAGCTCAATG ACCGTGTACA  1041 AGCCTGGAAT GAACAGACAC AACGGGAAAT GGGCTCACTG CCTCCGACCC CGACAACACC GGCCACCACC GACCAGCTCG  1121 CTGTTTGA  >> |
