# Supplementary material for: The Systematic Investigation of the Quorum Sensing System of the Biocontrol Strain Pseudomonas chlororaphis subsp. aurantiaca PB-St2 Unveils aurI to Be a Biosynthetic Origin for 3-Oxo-Homoserine Lactones
Source: PLoS One. 2016 Nov 18;11(11):e0167002. doi: 10.1371/journal.pone.0167002 (PMC5115851; doi:10.1371/journal.pone.0167002)
Supplement: S3 Table — (DOCX) [file pone.0167002.s011.docx]

**S3 Table. Nucleotide Sequence of *aurI* from *P. chlororaphis* subsp. *aurantiaca* StFRB508.**

| *aurI*_StFRB508_PciI*_lac*-promotor: |
| --- |
| 1 AAAAAAACAT GTTCTTTCCT GCGTTATCCC CTGATTCTGT GGATAACCGT ATTACCGCCT TTGAGTGAGC TGATACCGCT  81 CGCCGCAGCC GAACGACCGA GCGCAGCGAG TCAGTGAGCG AGGAAGCGGA AGAGCGCCCA ATACGCAAAC CGCCTCTCCC  161 CGCGCGTTGG CCGATTCATT AATGCAGCTG GCACGACAGG TTTCCCGACT GGAAAGCGGG CAGTGAGCGC AACGCAATTA  241 ATGTGAGTTA GCTCACTCAT TAGGCACCCC AGGCTTTACA CTTTATGCTT CCGGCTCGTA TGTTGTGTGG AATTGTGAGC  >> *lac*-promotor >> >> *lac*-  321 GGATAACAAT TTCACACAGG AAACAGCTAT GGAATTTATC GAATTTCACA CGCTCGACTA TAGTGCGACG CCCCACGCCT  operator >> >> *aur*I  401 GGGTCGCCGA TTTGTATGGC CTGCGCAAGG AAGTATTCGC CGATCGTTTG AACTGGAAGG TTAATATAAA GAATGACATC  481 GAGTTCGATG AGTACGACAA CGAGCGCACC ACCTACCTGA TCGGTACCTG GAAAGGCGTG CCCCTGGCCG GCCTGCGCCT  561 GATCAACACC CTGGATCCCT ACATGGTCGA AGGGCCGTTC CGCGACTTTT TCCGCTGCGC GCCGCCCAAG CAGGCGTTGA  641 TGGCTGAATC CAGCCGCTTT TTCGTCGACA AGACCCGCTC GCGCCAGCTC GGCCTGGCCC ATCTGCCGCT GACCGAAATG  721 CTCCTGTTGT GCATGCACAA CCATGCCGCG CGCAGCGGCC TGGAATCGAT CATCACGGTG GTCAGCAACG CCATGGGACG  801 GATCGTCCGC AATGCCGGCT GGCACTACGA AGTAATGGAC AGCGGCGAGG CCGCGCCGGG AGAAAAGGTG CTGTTGCTGA  881 ACATGCCGAT CAGCGACGCC AATCGTCAGC GCCTGCTGTC CAGCATCGCT CGCAAATGCC CCTTGTCATC CGCGCAGCTC  961 AACTCCTGGC CGCAGCGCCT GAACCCGCTC CACACAGCGC TCTACGAGCC GCAGCGGATT AGCGCATGA  >> |
