# Supplementary material for: The Systematic Investigation of the Quorum Sensing System of the Biocontrol Strain Pseudomonas chlororaphis subsp. aurantiaca PB-St2 Unveils aurI to Be a Biosynthetic Origin for 3-Oxo-Homoserine Lactones
Source: PLoS One. 2016 Nov 18;11(11):e0167002. doi: 10.1371/journal.pone.0167002 (PMC5115851; doi:10.1371/journal.pone.0167002)
Supplement: S4 Table — (DOCX) [file pone.0167002.s012.docx]

**S4 Table. Calibration equation and limit of quantification used for quantification of the corresponding AHLs.**

| AHL | calibration equation | R^2^ | limit of quantification [nM] |
| --- | --- | --- | --- |
| C4-HSL | y=0.00165*x-0.02278 | 0.995 | 0.37 |
| C6-HSL | y=0.00184*x-0.07777 | 0.998 | 1.51 |
| C8-HSL | y=0.00186*x+0.0249 | 0.997 | 0.44 |
| 3-OH-C6-HSL | y=0.00335*x-0.01442 | 0.995 | 0.23 |
| 3-OH-C8-HSL | y=0.00362*x-0.10924 | 0.992 | 0.82 |
| 3-OH-C10-HSL | y=0.00328*x-0.069 | 0.999 | 0.55 |
| 3-oxo-C6-HSL | y=0,00301*x+0.04865 | 0.997 | 0.47 |
| 3-oxo-C8-HSL | y=0.00293*x+0.00893 | 0.999 | 0.62 |

Only equations with a coefficient of determination (R^2^) > 0.99 were considered as sufficient for quantification. The limit of quantification represents the minimum amount that can be quantified in *P. aurantiaca* PB-St2 cultures.
