# Supplementary material for: The Systematic Investigation of the Quorum Sensing System of the Biocontrol Strain Pseudomonas chlororaphis subsp. aurantiaca PB-St2 Unveils aurI to Be a Biosynthetic Origin for 3-Oxo-Homoserine Lactones
Source: PLoS One. 2016 Nov 18;11(11):e0167002. doi: 10.1371/journal.pone.0167002 (PMC5115851; doi:10.1371/journal.pone.0167002)
Supplement: S5 Table — (DOCX) [file pone.0167002.s013.docx]

**S5 Table. Measured pH values of *P. aurantiaca* PB-St2 culture supernatants at different growth intervals.**

| t [h] | pH |
| --- | --- |
| 0 | 6.31 ± 0.01 |
| 5 | 6.31± 0.01 |
| 7 | 6.34 ± 0.01 |
| 9 | 6.45 ± 0.01 |
| 11 | 6.52 ± 0.01 |
| 13 | 6.57 ± 0.01 |
| 15 | 6.62 ± 0.01 |
| 17 | 6.64 ± 0.02 |
| 19 | 6.67 ± 0.03 |
| 21 | 6.75 ± 0.02 |
| 23 | 6.78 ± 0.01 |
| 25 | 6.86 ± 0.01 |
| 27 | 6.92 ± 0.01 |
| 42 | 7.10 ± 0.01 |

Data represent means with corresponding standard deviation of three independent replicates.
