# Supplementary material for: The Systematic Investigation of the Quorum Sensing System of the Biocontrol Strain Pseudomonas chlororaphis subsp. aurantiaca PB-St2 Unveils aurI to Be a Biosynthetic Origin for 3-Oxo-Homoserine Lactones
Source: PLoS One. 2016 Nov 18;11(11):e0167002. doi: 10.1371/journal.pone.0167002 (PMC5115851; doi:10.1371/journal.pone.0167002)
Supplement: S6 Table — (DOCX) [file pone.0167002.s014.docx]

**S6 Table. Sequence alignment of AurI.**

| AurI-StFRB508 1 MEFIEFHTLD YSATPHAWVA DLYGLRKEVF ADRLNWKVNI KNDIEFDEYD 50  \|\|.\|\|\|\|.\|\| \|\|\|\|\|\|\|\|\|\| \|\|:\|\|\|\|\|\|\| \|\|\|\|\|\|\|\|\|\| \|\|\|\|\|\|\|\|\|\|  AurI-PB-St2 1 MESIEFHALD YSATPHAWVA DLHGLRKEVF ADRLNWKVNI KNDIEFDEYD 50  AurI-StFRB508 51 NERTTYLIGT WKGVPLAGLR LINTLDPYMV EGPFRDFFRC APPKQALMAE 100  \|\|\|\|\|\|\|\|\|\| \|\|\|\|\|\|\|\|\|\| \|\|\|\|\|\|\|\|\|\| \|\|\|\|\|\|\|\|\|\| .\|\|\|\|\|\|\|\|\|  AurI-PB-St2 51 NERTTYLIGT WKGVPLAGLR LINTLDPYMV EGPFRDFFRC EPPKQALMAE 100  AurI-StFRB508 101 SSRFFVDKTR SRQLGLAHLP LTEMLLLCMH NHAARSGLES IITVVSNAMG 150  \|\|\|\|\|\|\|\|\|\| \|\|\|\|\|\|\|\|\|\| \|\|\|\|\|\|\|\|\|\| \|\|\|\|\|\|\|\|\|\| \|\|\|\|\|\|:\|\|\|  AurI-PB-St2 101 SSRFFVDKTR SRQLGLAHLP LTEMLLLCMH NHAARSGLES IITVVSSAMG 150  AurI-StFRB508 151 RIVRNAGWHY EVMDSGEAAP GEKVLLLNMP ISDANRQRLL SSIARKCPLS 200  \|\|\|\|\|\|\|\|\|\| \|\|\|\|:\|\|\|\|\| \|\|\|\|\|\|\|\|\|\| \|\|\|\|\|\|\|\|\|\| \|\|\|\|\|\|\|\|\|\|  AurI-PB-St2 151 RIVRNAGWHY EVMDTGEAAP GEKVLLLNMP ISDANRQRLL SSIARKCPLS 200  AurI-StFRB508 201 SAQLNSWPQR LNPLHTALYE PQRISA 226  \|\|\|\|\|.\|\|\|\| \|\|\|\|\|\|\|\|.\| \|\|\|\|\|\|  AurI-PB-St2 201 SAQLNHWPQR LNPLHTALCE PQRISA 226 |
| --- |

“|” indicates positions which have a single, fully conserved residue. “:” indicates conservation between groups of strongly similar properties. “.”indicates conservation between groups of weakly similar properties. The yellow color highlights the eight different amino acids. GenBank accession numbers for the protein sequences: AurI-StFRB508: BAM94429.1, AurI-PB-St2: ETD40527.1.
